# Incorporating time-delays in S-System model for reverse engineering genetic networks

**DOI:** 10.1186/1471-2105-14-196

**Published:** 2013-06-18

**Authors:** Ahsan Raja Chowdhury, Madhu Chetty, Nguyen Xuan Vinh

**Affiliations:** 1 Gippsland School of Information Technology, Monash University, Churchill, Victoria-3842, Australia; 2 National Information and Communication Technology Australia (NICTA), Melbourne, Australia

## Abstract

**Background:**

In any gene regulatory network (GRN), the complex interactions occurring amongst transcription factors and target genes can be either *instantaneous* or *time-delayed*. However, many existing modeling approaches currently applied for inferring GRNs are unable to represent both these interactions simultaneously. As a result, all these approaches cannot detect important interactions of the other type. S-System model, a differential equation based approach which has been increasingly applied for modeling GRNs, also suffers from this limitation. In fact, all S-System based existing modeling approaches have been designed to capture *only* instantaneous interactions, and are unable to infer time-delayed interactions.

**Results:**

In this paper, we propose a novel Time-Delayed S-System (TDSS) model which uses a set of delay differential equations to represent the system dynamics. The ability to incorporate time-delay parameters in the proposed S-System model enables simultaneous modeling of both instantaneous and time-delayed interactions. Furthermore, the delay parameters are not limited to just positive integer values (corresponding to time stamps in the data), but can also take fractional values. Moreover, we also propose a new criterion for model evaluation exploiting the sparse and scale-free nature of GRNs to effectively narrow down the search space, which not only reduces the computation time significantly but also improves model accuracy. The evaluation criterion systematically adapts the max-min in-degrees and also systematically balances the effect of network accuracy and complexity during optimization.

**Conclusion:**

The four well-known performance measures applied to the experimental studies on synthetic networks with various time-delayed regulations clearly demonstrate that the proposed method can capture both instantaneous and delayed interactions correctly with high precision. The experiments carried out on two well-known real-life networks, namely IRMA and SOS DNA repair network in *Escherichia coli* show a significant improvement compared with other state-of-the-art approaches for GRN modeling.

## Introduction

The availability of genome wide expression data has significantly increased interest in systems biology, in particular, reverse-engineering gene regulatory networks (GRNs). While static expression data allows the learning of only the network structure, i.e., transcription factors (TF) and target genes interactions, time-course data allows the modeling of detailed system dynamics over time. In our view, amongst different ways for classification [1-5], methods for reverse-engineering GRNs can be broadly categorized into six major groups, namely (i) co-expression network, (ii) Bayesian network, (iii) differential equation based approach, (iv) regression based approach, (v) meta approaches combining two or more different methods, and (vi) approaches that are based on other principles. Co-expression networks [6,7] are coarse-scale, simplistic models that employ pairwise association measures, such as the partial correlation or conditional mutual information, for inferring the interactions between genes. These methods have low computational complexity and thus can easily scale up to very large networks of thousands of genes [8], but lack a mechanism for modeling system dynamics. Bayesian networks (BN) are more sophisticated models based on the strong foundation of probability and statistics, in which the dependencies between nodes are represented using directed edges and conditional probability distributions. A temporal form of BN, i.e., dynamic Bayesian network (DBN), allows the modeling of system dynamics in discrete time.

In this paper, we focus on differential equation (DE) based approaches, which belong to a sophisticated and well established class of methods for modeling biochemical phenomena, including GRNs [9-13]. A salient feature of all DE-based approaches is their ability to accurately model system dynamics in *continuous* time. Of the several linear and non-linear types of DE models employed for reconstructing GRNs, the S-System model has gained popularity recently [14-19]. Originating from the pioneering work of Savageau [20], the S-System has been considered as an excellent balance between model complexity and mathematical tractability: it is complex enough to represent a wide range of dynamics, yet is simple enough to allow certain analytical studies.

**Figure 1:**
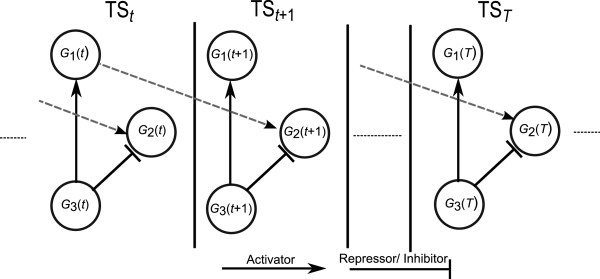
**Instantaneous and delayed interactions among genes in an illustrative 3-gene network having a total of *T *data points.***G*_*i*_(*t*) represents the *i*^*th*^ gene in *TS*_*t*_ time interval, solid lines represent instantaneous interactions (both activator and repressor) and dotted lines represent time-delayed interaction.

In GRNs, almost all genetic interactions are invariably delayed. Furthermore, these delayed interactions may have different time lags [21]. Time delays in regulatory interactions are due to the time required for the regulator gene to express its protein product and for the transcription of the target genes to be affected by these regulatory proteins. More specifically, this is the time required for the translation, folding, nuclear translocation, turnover for the regulatory protein, and elongation of the target gene mRNA. For example, in mammals, the transcriptional regulatory time-delays can be from several minutes to several tens of minutes, and are composed of two components: the TF translation/post-translational processing/translocation time (∼10.5±4 mins), and the target gene transcription and post-transcription processing time (∼20-40 mins) [22]. The instantaneous and time-delayed interactions among genes in a toy 3-gene network are illustrated in Figure 1. As we can see, gene *G*_3_ instantaneously regulates genes *G*_1_ and *G*_2_, while *G*_1_ has a 1-unit-time delayed regulation on *G*_2_.

Most existing approaches for modeling GRNs attempt to capture instantaneous (non-temporal) interactions only. This is the case for all co-expression based approaches and static Bayesian networks, which do not differentiate between static and time-course expression data. There have been previous attempts for modeling time-delayed genetic interactions with dynamic Bayesian network using time-course data, such as the method proposed by Zou and Conzen [21], and also our recently proposed method GlobalMIT [23] and [24] (which we call BITGRN2 throughout this paper, as [24] is the improved version of BITGRN). The Recursive Neural Network (RNN) based methods [25-29], capable of interpreting complex temporal behavior of gene expression data, have the ability to work with time-delays. However, this time-delay issue is either not well-addressed [28,29] or the delays are fixed for most of the existing approaches [25-27]. Further, so far RNN based methods are incapable of presenting regulations in the degradation phase, which is an inherent feature of S-System model. The ordinary differential equation (ODE) based methods [9-11] are limited to work with instantaneous interactions and are incapable of inferring time-delayed regulations. On the other hand, a delay differential equations (DDE) based model was employed in [12], that works with delay, but the time delay parameters were set manually rather than via learning from data. Kim *et al.*[13] proposed a DDE based method that is capable of working with time-delays. However, the method is limited to working with fixed delays, which are either set to their a-priori known values, or otherwise initialized randomly then fixed during the optimization. To the best of our knowledge, there is no differential equation based approach available that can model time-delayed and instantaneous interactions simultaneously, with the flexibility to adapt the delay parameters through optimization.

The main contributions of this paper are two-fold. First, it proposes a novel time-delayed S-System model based on a set of delay differential equations (DDE) which is capable of simultaneously capturing *both* – time-delayed and instantaneous interactions. Further, it incorporates time delay parameters which are not restricted to take only integer values (corresponding to time stamps in the data) as possible in other discrete-time approaches (e.g., dynamic BN), but they can take fractional values. This allows the model to capture the time delays in genetic interactions with higher accuracy, because in reality, the amount of delay takes continuous value. Second, to overcome the limitations of previous optimization approaches, our new search algorithm is designed systematically exploiting the sparse and scale-free nature of GRNs to effectively narrow down the search space. Compared to the existing two S-System based modeling approaches [16,19], the proposed approach learns the parameters more accurately despite an increase in the number of model parameters to be learnt. Experimental studies on two synthetic and two real genetic networks show a significant improvement over recently proposed modeling techniques.

## Background

### Traditional S-System model

For a network of *N* genes, the existing S-System model is given by the following set of ordinary differential equations (ODEs):

(1) $$\frac{{\text{dX}}_{i}}{\text{dt}}=\alpha_{i}\overset{N}{\underset{j=1}{\prod }}X_{j}^{g_{\text{ij}}n}-\beta_{i}\overset{N}{\underset{j=1}{\prod }}X_{j}^{h_{\text{ij}}},\;i=1\ldots N$$

Here, for any *i*^*th*^ gene, *X*_*i*_ is the expression level, {*α*_*i*_,*β*_*i*_}’s are the rate constants, and { *g*_*ij*_,*h*_*ij*_}’s are the kinetic orders. The term $\alpha_{i}\prod \overset{g_{\text{ij}}}{\underset{j}{X}}$ models the process of RNA *synthesis/production*, while the term $\beta_{i}\prod \overset{h_{\text{ij}}}{\underset{j}{X}}$ models the process of RNA *degradation*. To infer a GRN of *N* genes using the S-System model, 2*N* (*N* + 1) parameters must be estimated. To reduce computational complexity, method of [30] approximated the original problem as *N* decoupled sub-problems, each having 2(*N* + 1) parameters. In the *i*^*th*^ sub-problem corresponding to the *i*^*th*^ gene, the parameter set *Ω*_*i*_ = {*α*_*i*_,*β*_*i*_,{*g*_*ij*_,*h*_*ij*_}_*j* = 1 …*N*_} is estimated by solving the following decoupled S-System equation:

(2) $$\frac{{\text{dX}}_{i}}{\text{dt}}=\alpha_{i}\overset{N}{\underset{j=1}{\prod }}Y_{j}^{g_{\text{ij}}}-\beta_{i}\overset{N}{\underset{j=1}{\prod }}Y_{j}^{h_{\text{ij}}}$$

For solving Eqn. (2), only *Y*_*i*_ = *X*_*i*_ is computed by numerical integration, while $Y_{j}={\hat{X}}_{j},\forall j\neq i$, where ${\hat{X}}_{j}$ is obtained by a direct estimation based on the observed expression data of the *j*^*th*^ gene [15]. For direct estimation, the commonly used technique of linear spline interpolation [31] can be applied. Although this approximation may decrease the accuracy slightly, the significantly reduced computational burden allows the optimization process to converge to better solutions in much shorter time.

### Model evaluation criteria

In order to assess the goodness of S-System models, previous works commonly employed the squared relative error (SRE) as criterion for model evaluation. As the parameters for each gene in the decoupled systems are learned independently of the others, the SRE for *i*^*th*^ gene is given as:

(3) $$\text{SRE}=\overset{T}{\underset{t=1}{\sum }}{\left(\frac{X_{i}^{\text{cal}}(t)-X_{i}^{\text{exp}}(t)}{X_{i}^{\text{exp}}(t)}\right)}^{2}$$

Here *t* denotes a specific time-stamp (TS) in the observed time series of *T* sample points. $X_{i}^{\text{cal}}(t)$ and $X_{i}^{\text{exp}}(t)$ denote the calculated and observed expression value of gene *i* at time-stamp *t* respectively. It is to be noted that if the data set consists of several separate time series, then the SRE criterion can simply be extended by summing over all the available time series. Due to decoupling, this SRE criterion for each gene can be minimized independently. The solution for this optimization problem is normally dense, i.e., it has many non-zero parameter values corresponding to many regulators for each gene. However, it is widely reported that GRNs are sparse in nature, and in fact follow a scale-free topology [32,33]. Thus, a regularization term, similar to LASSO regression, is often added. Authors of [15] were the first to propose a penalty term for model complexity (Eqn. (1) of the supplementary document (Additional file 1)), which was subsequently improved by Noman and Iba [17,34] as follows:

(4) $$\text{RSRE}=\overset{T}{\underset{t=1}{\sum }}{\left(\frac{X_{i}^{\text{cal}}(t)-X_{i}^{\text{exp}}(t)}{X_{i}^{\text{exp}}(t)}\right)}^{2}+c\overset{2N-I}{\underset{j=1}{\sum }}(|K_{\text{ij}}|)$$

with *K*_*ij*_ being the kinetic orders of gene-*i* sorted in ascending absolute values, *I* being the maximum number of regulators allowed for each gene, and *c* being the penalty constant. In this paper, we are referring to Eqn. (4) as regularized squared relative error (RSRE) as it is essentially a regularized version of Eqn. (3). The limitations of the RSRE criterion are: i) although promoting sparse solutions, it still encourages every gene to take on several regulators, since up to *I* regulators can be taken, free of any penalty, ii) *I* is a global parameter applied to all genes. Since the number of potential regulators for different genes are different, it is preferable to have the maximum in-degree parameter being set adaptively and specifically for each gene, and iii) the penalty weight *c* is fixed, and thus there is no mechanism for dynamically prioritizing its two objectives (i.e., the two RHS terms) during the optimization. This prioritization is important because, for example, during initial stages of optimization, it is necessary to have emphasis on reducing error, i.e., improving model accuracy (first term), while in the later stages, the emphasis shifts towards reducing model complexity (second term). Our recently proposed evolutionary search [19], unlike previous methods with fixed *I*, was able to continuously adjust both the value of *I* (max in-degree) and *J* (min in-degree) for every gene based on population statistics:

(5) $${\text{RSRE}}_{2}=\overset{T}{\underset{t=1}{\sum }}{\left(\frac{X_{i}^{\text{cal}}(t)-X_{i}^{\text{exp}}(t)}{X_{i}^{\text{exp}}(t)}\right)}^{2}+C_{i}\frac{2N}{Z_{\text{Count}}}$$

Here, *Z*_*Count*_ is the total number of non-regulations for the *i*^*th*^ gene ( = 2*N* - total regulations) and, *C*_*i*_ is the scaling factor for the *i*^*th*^ gene defined as:

(6) $$C_{i}=\left\{\begin{array}{ll} 1 & \text{if}\;I\geq r_{i}\geq J\;\text{or}\;r_{i}=0\\ \frac{J}{r_{i}}2^{\left(J-r_{i}\right)} & \text{if}\;r_{i}<J\\ \frac{r_{i}}{I}2^{\left(r_{i}-I\right)} & \text{if}\;r_{i}>I \end{array}\right)$$

Here, *r*_*i*_ is the number of transcription factors (total regulations) for gene-*i*. Details about this fitness function are available in [19] and a brief discussion is included in Section 1.2 of the supplementary document (Additional file 1). Although, the penalty graph generated by the model complexity part resembles the property of power-law formalism, the addition of another fractional term in the prefixes of the exponential term (*J*/*r*_*i*_ and *r*_*i*_/*I*) makes the penalty term asymmetric. While a preliminary study [19] on this fitness criteria showed improvement, the applied penalty function being adhoc is not well justified.

## Methods

### The proposed time-delayed S-System model

#### ***Modeling time-delayed interactions***

The traditional S-System described in Eqn. (1) is a set of ordinary differential equations (ODE), in which the rate of change of a gene expression at a specific time instant *t* is affected by its own and all other genes’ expression values at that instant. In other words, the model is not versatile to capture time delayed interactions which invariably occur in all biological systems. To do this, it is necessary to replace the system dynamics represented by ODE in Eqn. (1) with delay differential equations (DDE). Let us consider a DDE of the following form:

(7) $$\dot{x}=f(x(t-\tau ))$$

with the delay parameter *τ* ∈ [ 0,*∞*). However, as the rate of change of system response is affected by its previous values at time (*t* - *τ*), in practice, *τ*∈ [ 0,*t*_*max*_), where *t*_*max*_ is the time-span of the microarray time series experiment. The Time-Delayed S-System (TDSS) model with a single and fixed delay (*τ*) can be represented as follows:

(8) $$\frac{{\text{dX}}_{i}}{\text{dt}}=\alpha_{i}\overset{N}{\underset{j=1}{\prod }}X_{j,t-\tau }^{g_{\text{ij}}}-\beta_{i}\overset{N}{\underset{j=1}{\prod }}X_{j,t-\tau }^{h_{\text{ij}}},\;i=1\ldots N$$

In S-System models with *N* genes, there can be 2 × *N* × *N* regulations, where any of them can be a time-delayed regulation. Hence, we generalize Eqn. (8) in the following form: ?

(9) $$\frac{{\text{dX}}_{i}}{\text{dt}}=\alpha_{i}\overset{N}{\underset{j=1}{\prod }}X_{j,t-\tau_{\text{ij}}^{g}}^{g_{\text{ij}}}-\beta_{i}\overset{N}{\underset{j=1}{\prod }}X_{j,t-\tau_{\text{ij}}^{h}}^{h_{\text{ij}}},\;i=1\ldots N$$

Here, $X_{j,t-\tau_{\text{ij}}}$ denotes the value of gene *X*_*j*_ at time *t* - *τ*_*ij*_, with the delay parameter *τ*_*ij*_∈ [ 0,*τ*_*max*_], where *τ*_*max*_ is the maximum possible delay in the considered network. Note that there are two time delay parameters: $\tau_{\text{ij}}^{g}$ corresponding to the *production* part and $\tau_{\text{ij}}^{h}$ corresponding to the *degradation* part of S-System. The delay matrices are represented as follows:

(10) $$\tau^{g}=\left(\begin{array}{llll} \tau_{1,1}^{g} & \tau_{1,2}^{g} & \cdots & \tau_{1,N}^{g}\\ \tau_{2,1}^{g} & \tau_{2,2}^{g} & \cdots & \tau_{2,N}^{g}\\ \vdots & \vdots & \ddots & \vdots \\ \tau_{N,1}^{g} & \tau_{N,2}^{g} & \cdots & \tau_{N,N}^{g} \end{array}\right)$$

(11) $$\tau^{h}=\left(\begin{array}{llll} \tau_{1,1}^{h} & \tau_{1,2}^{h} & \cdots & \tau_{1,N}^{h}\\ \tau_{2,1}^{h} & \tau_{2,2}^{h} & \cdots & \tau_{2,N}^{h}\\ \vdots & \vdots & \ddots & \vdots \\ \tau_{N,1}^{h} & \tau_{N,2}^{h} & \cdots & \tau_{N,N}^{h} \end{array}\right)$$

For both these matrices, {$0\leq \{\tau_{i,j}^{g},\tau_{i,j}^{h}\}\leq \tau_{\text{max}}$}, ∀_*i*,*j* = 1 …*N*_ and *τ*_*max*_ is the maximum allowed delay of the network.

At any time, the production and degradation rate of the *i*^*th*^ gene is affected by its own and other genes’ concentration level at their corresponding delays. If a delay *τ*_*ij*_, corresponding to an interaction (*g*_*ij*_/ *h*_*ij*_), is 0, we have an instantaneous interaction (provided that there is a regulation between genes *i* and *j*), whereas a non-zero value of *τ*_*ij*_ gives a delayed interaction. Thus, the proposed Time-Delayed S-System (TDSS) model is capable of capturing both time delayed and instantaneous genetic interaction in GRNs.

#### ***Model parameters***

For the traditional S-System model, in the *i*^*th*^ sub-problem corresponding to the *i*^*th*^ gene, the 2*N* + 2-parameter set *Ω*_*i*_ = {*α*_*i*_,*β*_*i*_,{*g*_*ij*_,*h*_*ij*_}_*j* = 1…*N*_} needs to be estimated. In the Time-Delayed S-System model, apart from these parameters, we also have to estimate the 2*N* time-delay parameters ${\left\{\tau_{\text{ij}}^{g},\tau_{\text{ij}}^{h}\right\}}_{j=1\ldots N}$. Thus, a 4*N* + 2-parameter set $\Omega_{i}=\{\alpha_{i},\beta_{i},{\left\{g_{\text{ij}},h_{\text{ij}},\tau_{\text{ij}}^{g},\tau_{\text{ij}}^{h}\right\}}_{j=1\ldots N}\}$ needs to be learned. For learning the time-delay parameters, we follow a two-stage approach. First, we employ the Pearson correlation coefficient (PCC) technique to identify the most probable lag of the interaction between any pair of genes. For doing this, we use linear spline interpolation to intrapolate additional data points between any two actual measurements. For a given data set, the maximum time delay (*τ*_*max*_) permissible for the system is set by considering common regulation time scale (ranging within tens of minutes [22]) and the data sampling rate. Although the proposed TDSS is capable of dealing with any resolution of fractional delay, in this paper we have limited the minimum time-delay step size to 1/10 of the time between two time-stamps, provided that the data are regularly sampled. Else, the time-delay step size is set to 1/10 of the time between two closest time-stamp in non-regularly sampled data. While using PCC, we fix the expression profile of a regulator gene and shift the target gene’s expression profile forward incrementally one step at a time (minimum time-delay step). The time lag maximizing PCC is considered as the most probable time lag between these two genes. These most probable lag values are then used to initialize the time delay parameters for the evolutionary optimization phase.

#### ***Time responses***

In the traditional S-System model, numerical integration is normally performed with the well-known fourth order Runge-Kutta method (RK4). For the Time-Delayed S-System model, we adapt the traditional RK4 method for DDE which takes into account the time delay parameters as described in detail in [35]. For the adapted RK4, initial samples of the regulator gene’s expression profile of length *τ*_*max*_ will be designated as history information, which reduces the available sample size for training. It should be noted that the step size for RK4 integration is set at a small value, allowing the numerical integration to capture the system dynamics accurately. Again, we use linear spline interpolation to generate a continuous history profile. A detailed description of the modified RK4 is presented in Sec. 2.3 in the supplementary document (Additional file 1).

### Inference mechanism

Due to the intractable nature of optimization problem, S-System parameter learning is commonly carried out via evolutionary computation (EC), namely Genetic Algorithm (GA) or Differential Evolution (DE). Recently, DE and its variants, such as trigonometric differential evolution (TDE), have been used extensively because of their versatility [18,19,36-38]. As an optimization tool for learning model parameters, both DE and TDE perform better than the other conventional evolutionary computation approaches [18,19]. In this paper, we employ a new TDE approach for learning TDSS parameters. We also employ the Multistage Refinement Algorithm (MRA) [19] as a pruning mechanism for eliminating the weak regulations from the resulting network. Details related to TDE, initial population generation, and MRA are presented in Sec. 1.3 and Sec. 2.4 of the supplementary document (Additional file 1).

#### ***Model evaluation criterion***

To address various limitations of the regularized squared relative error of Eqn. (4) presented in Sec. 2, we propose a novel fitness function referred to as *adaptive squared relative error* (ASRE) and given below:

(12) $$\text{ASRE}=\overset{T}{\underset{t=1}{\sum }}{\left(\frac{X_{i}^{\text{cal}}(t)-X_{i}^{\text{exp}}(t)}{X_{i}^{\text{exp}}(t)}\right)}^{2}+B_{i}\times C_{i}\frac{2N}{2N-r_{i}}$$

Here, *r*_*i*_ is the total number of actual regulators. *B*_*i*_ is a balancing factor which is used to maintain desired balance between the two terms of ASRE. *C*_*i*_ is the penalty factor for the *i*^*th*^ gene, defined as:

(13) $$C_{i}=\left\{\begin{array}{ll} 1 & \text{if}\;J<r_{i}<I\\ 1+{\left(J-r_{i}\right)}^{2} & \text{if}\;r_{i}\leq J\\ 1+{\left(r_{i}-I\right)}^{2} & \text{if}\;r_{i}\geq I \end{array}\right)$$

with *I* and *J* being the maximum and minimum in-degree respectively. Note that in our formulation, *r*_*i*_ and *I* are restricted to be smaller than or equal to *N*, since a transcription factor generally does not affect both its target gene’s production and degradation simultaneously. In our ASRE criterion, in contrast to a *fixed* weighting factor *c* as in Eqn. (4), the penalty factor *C*_*i*_ takes the form of an inverse power-law function. This is motivated by the fact that biological networks often have a scale-free structure, in which the node connectivity degree *x* distributes according to a power-law distribution, *P*(*x*) ∝ *x*^-*γ*^, with the scaling parameter *γ* ∈ [ 2,3] for various networks in nature, society and technology [33]. Gene regulatory networks generally have low in-degrees, with the number of genes having high in-degree diminishing according to a power-law form. Note that in our formulation, we also enforce a minimum in-degree *J*, thus genes with the number of in-degree falling in-between the min-max number of in-degree [*J*,*I*] are not penalized (*C*_*i*_ = 1), while genes falling out of this region are penalized according to an inverse power law term (*C*_*i*_ = 1 + *d*^*γ*^, where *γ* = 2 and *d* is the number of missing or violated regulations). Sec. 2.4.2 and 2.4.3 in the supplementary document (Additional file 1) explain how our algorithm adaptively adjusts the [*J*,*I*] region during the optimization process.

#### ***Salient features***

We highlight the salient features of the proposed optimization framework as follows: 

***(i) Adaptive regulator set size: *** Our algorithm adaptively and continually adjusts the values of the min-max in-degree region [*J*,*I*]. Initially, we set *J* = 0 and *I* = a value less than or equal to *N* based on the size of the network. Then, for every *l* generations, we examine the smallest and largest in-degree within the population respectively and set these as new values for *J* and *I*.

***(ii) Adaptive balancing factor ****B*_*i*_: The balancing factor *B*_*i*_ is included in Eqn. (12) to dynamically balance the terms corresponding to the network accuracy and the model complexity. For the first initial tens of generations, we set the value of *B*_*i*_ to zero, i.e., we emphasize on network quality first. This allows the algorithm to quickly improve the network accuracy as there are no constraints on complexity. We allow the algorithm to proceed in this manner either until a fixed *n*_*e*_ generations are executed or until the squared relative error is smaller than a specified threshold *γ*_*i*_. When the individuals in the population achieve stability and improved accuracy, the value of *B*_*i*_ is updated as follows: from the top 50% individuals in the population, we calculate the average network accuracy ANA (first term of Eqn. (12)) and the average model complexity AMC (second term of Eqn. (12), i.e., 2*N*/(2*N*-*r*_*i*_)), then set *B*_*i*_ = *ANA*/*AMC*. With this, effect of the network accuracy is maintained in ‘balance’ with model complexity. Next, we replace the worst 50% individuals with randomly initialized individuals, and the optimization continues with the value of *B*_*i*_ computed as above.

While our preliminary studies reported earlier [19] also used adaptation of *I* and *J*, the implementation was rather adhoc, and had static weight factor. The proposed model evaluation criteria represented by Eqn. (12) and Eqn. (13) are thus novel and perform systematic adaptation of *I* and *J* while also simultaneously carrying out adaptive balancing of network complexity and accuracy.

## Results and discussions

The proposed TDSS model is evaluated experimentally using both synthetic and real-life networks. As the model parameters increase quadratically with the network size, large scale modeling with the S-System based models remains a long-standing challenge. For this reason, like previous research on the S-System [16,19,39-41], we mainly test our method on small and medium sized networks. We employ two synthetic network studies of different sizes, i.e., a small network with 5 genes and a 20-gene medium sized network. For real-life network studies, we present experiments on two small networks, namely IRMA that contains 5 genes, and SOS DNA Repair Network in *Escherichia coli* containing 8 genes.

With synthetic networks, we investigate network configurations having no delays (instantaneous interactions only) and also in the presence of delays (both instantaneous and time-delayed). For each of these configurations, along with noise free data, we have considered three different levels of Gaussian noise. The four well-known performance measures [24,42] namely sensitivity (*S*_*n*_), specificity (*S*_*p*_), precision (*P*_*r*_) and F-score (*F*) have been applied for network evaluation. For the methods with executable code available, namely ALG [16,34] and REGARD [19], we run the respective programs on our generated data. For other methods where no code is available, we extract the performance measure values from their respective original publications for comparison where possible.

With real-life networks, for IRMA, the comparison is carried out with 7 other approaches, namely, ALG [16,34], REGARD [19], BITGRN2 [24,42], BANJO [43], TDARACNE [44], NIR and TSNI [45], ARACNE [7]. For the *E. coli* network, the performance has been evaluated with ALG [34], REGARD [19], S-Tree based approach [39], two approaches from Kimura *et al.*[40,46], and several BN based approaches, e.g., [47], BANJO [43], BITGRN2 [24,42] and GlobalMIT [23]. In addition, the time-responses of the inferred networks are compared with the actual time expression profiles to show the accuracy of the proposed model in capturing gene expression dynamics. All the inferred time-responses are shown for the best inference result (in terms of the objective function value, out of 5 separate runs) with error bars indicating the 95% confidence interval (CI).

The proposed algorithm is implemented in C++ using a 2.16 GHz Dual-core CPU PC with 3 GB of RAM. This code is made available upon request. The parameter values for the TDE algorithm were set as follows: Mutation Factor *F*_*o*_ = 0.5, Trigonometric Mutation Factor *F*_*t*_ =0.05, Crossover Factor *CF* = 0.8, population size *Pop* = 100. The number of generations when *B*_*i*_ =0 is set to *n*_*e*_ =50 and the specified threshold *γ*_*i*_ to half the value of ASRE of the best individuals in initial population. Once *B*_*i*_ is reset, the in-degrees are updated with ARGC algorithm (details in Sec. 2.4.3 of Additional file 1) in every *l* = 50 generations. The pruning factor *ψ* = 0.25 (details in Sec. 2.4.5 of Additional file 1) is used in both the stages of Multistage Refinement Algorithm (MRA). For synthetic network, *M* = 10 datasets are used for reverse-engineering, generated for each network from 10 different initial conditions. We have executed the proposed optimization method with TDSS for 1000 generations in the first phase while in the the second phase, MRA is executed for 250 generations. The maximum time delay value (*τ*_*max*_) was set to 3 time-stamps (TS) for all the synthetic networks, as the maximum delay among all delayed regulations was manually set to 3TS for synthetic data generation. For the IRMA networks, *τ*_*max*_ was set to 100 minutes, equivalent to 10TS. For the *E. coli* network, we set *τ*_*max*_ to 1h, which is also 10TS. The experiments are carried out with 5 separate runs of TDSS with different random initializations for each network. In the following, the best case result represents the best solution of these 5 separate runs, in terms of the objective function value, i.e., the adaptive squared relative error (ASRE) in Eqn. (12).

### Synthetic networks

#### ***Small scale synthetic network***

**Figure 2:**
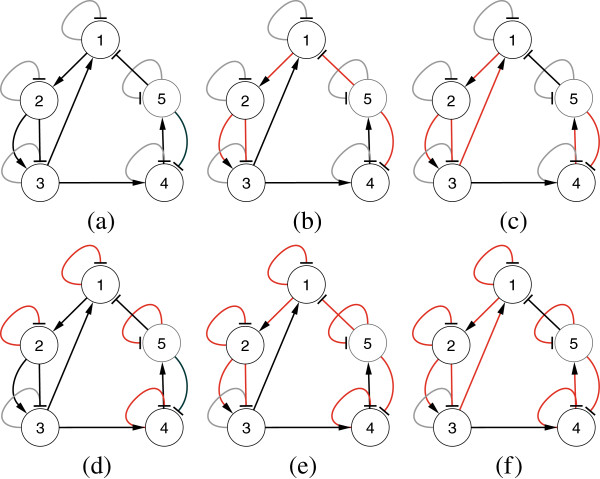
**All three configurations of 5-gene network, both target and inferred (a) Conf-1 (Target) (b) Conf-2 (Target) (c) Conf-3 (Target) (d) Conf-1 (Inferred) (e) Conf-2 (Inferred) (f) Conf-3 (Inferred).** Arrow ended black lines and block ended gray lines indicate instantaneous activation and suppression, respectively, while red lines indicate time-delayed regulations.

We investigate the widely studied 5-gene synthetic network, first proposed in [14], with the relevant network parameters given in Table 1. From this base network topology, we have generated three different configurations for testing: one network with no delay (Conf-1) and two with delays (Conf-2 and Conf-3). The networks for all three configurations are shown in Figure 2(a-c), while the time delay parameter values are shown in Table 2. In all three cases, we have evaluated the performance of TDSS with and without the presence of noise in data.

**Table 1:** **S-System parameters for the 5-gene synthetic network of [**[14]**]**

Gene 1	*α*_1_=5.0,*g*_1,3_=1.0,*g*_1,5_=-1.0,*β*_1_=10.0,*h*_1,1_=2.0
Gene 2	*α*_2_=10.0,*g*_2,1_=2.0,*β*_2_=10.0,*h*_2,2_=2.0
Gene 3	*α*_3_=10.0,*g*_3,2_=-1.0,*β*_3_=10.0,*h*_3,2_=-1.0,*h*_3,3_=2.0
Gene 4	*α*_4_=8.0,*g*_4,3_=2.0,*g*_4,5_=-1.0,*β*_4_=10.0,*h*_4,4_=2.0
Gene 5	*α*_5_=10.0,*g*_5,4_=2.0,*β*_5_=10.0,*h*_5,5_=2.0
Remaining *g*_*i*,*j*_=*h*_*i*,*j*_=0,∀*i*,*j* = 1,2…,5	

**Table 2:** Three different delay configurations of the 5-gene synthetic network

Configuration 1	$\tau_{i,j}^{g}=\tau_{i,j}^{h}=0$ (Non-delayed network)
(Conf-1)	∀*i*,*j* = 1,2…,5
Configuration 2	$\tau_{1,5}^{g}=\tau_{2,1}^{g}=\tau_{3,2}^{g}=\tau_{3,2}^{h}=\tau_{4,5}^{g}=1.0$
(Conf-2)	remaining $\tau_{i,j}^{g}=\tau_{i,j}^{h}=0,\forall i,j=1,2\ldots ,5$
Configuration 3	$$\tau_{1,3}^{g}=1.1,\tau_{2,1}^{g}=1.2,\tau_{3,2}^{g}=1.3,\tau_{5,4}^{g}=2.1$$ , $\tau_{4,5}^{g}=\tau_{3,2}^{h}=1.0$,
(Conf-3)	remaining $\tau_{i,j}^{g}=\tau_{i,j}^{h}=0,\forall i,j=1,\ldots ,5$

##### A. Network with no-delay (Conf-1)

**Figure 3:**
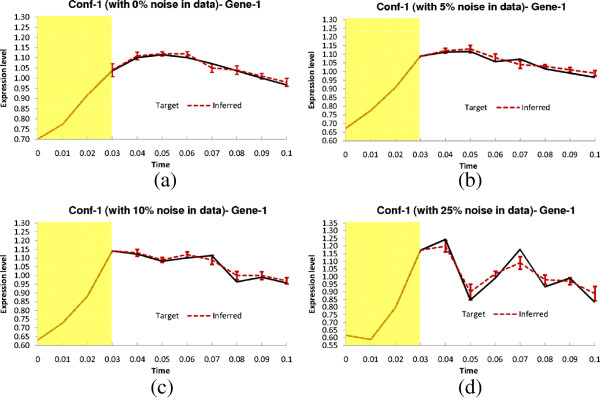
**Dynamics for Gene-1 of Conf-1.** Solid lines and dotted lines indicate respectively target and inferred (by TDSS) time-expressions in **(a)** Noise free data **(b)** 5% Noise in data **(c)** 10% Noise in data **(d)** 25% Noise in data. The yellow shaded region indicates the history information and the error bars indicate 95% confidence interval.

In this configuration, all the delay parameters are set to zero. In addition, the methods were also evaluated on noise-free data, as well as data generated with three different levels of Gaussian noise (5%, 10%, and 25%). The performance metrics (*S*_*n*_, *S*_*p*_, *P*_*r*_, *F*) for the non-delayed network in noise-free setting and with three different levels of noise are shown in Table 3. The proposed method successfully inferred all the regulations (*S*_*n*_ = 1) even in the presence of 25% noise level. Moreover, the other three metrics for TDSS are also very close to the optimal value. It should be noted that, compared to other methods reported in Table 3 the four performance metrics for TDSS are on par with several methods and better than most others. Figure 3(a-d) show the time-responses of a particular gene for all four levels of noise. In particular, we have selected gene-1, which exhibits significant changes in expression value over the time course. In addition to detecting all the regulations correctly, the inferred time-responses are very close to the target expressions. The time-responses for another gene (gene-2) along with the inferred parameters for TDSS (best case result for noise-free data) are shown in Sec. 3 in the supplementary document (Additional file 1). The inferred network for noise-free data (best case) is shown in Figure 2(d).

**Table 3:** Experimental results on Conf-1 (5-gene synthetic network)

	**Conf-1 (No-delay network)**															
	**0% Noise**				**5% Noise**				**10% Noise**				**25% Noise**			
	***S***_***n***_	***S***_***p***_	***P***_***r***_	***F***	***S***_***n***_	***S***_***p***_	***P***_***r***_	***F***	***S***_***n***_	***S***_***p***_	***P***_***r***_	***F***	***S***_***n***_	***S***_***p***_	***P***_***r***_	***F***
TDSS (Best)	1.00	1.00	1.00	1.00	1.00	1.00	1.00	1.00	1.00	0.95	0.87	0.93	1.00	0.87	0.72	0.84
TDSS	1.00 ±	1.00 ±	1.00 ±	1.00 ±	1.00 ±	0.98 ±	0.95 ±	0.97 ±	1.00 ±	0.93 ±	0.84 ±	0.91 ±	1.00 ±	0.84 ±	0.68 ±	0.81 ±
(Average ±Std)	0.00	0.00	0.00	0.00	0.00	0.02	0.05	0.03	0.00	0.02	0.05	0.03	0.00	0.01	0.02	0.01
ALG [34]	1.00	0.35	0.35	0.52	1.00	0.68	0.52	0.68	0.92	0.65	0.48	0.63	0.91	0.64	0.46	0.60
REGARD [19]	1.00	1.00	1.00	1.00	1.00	0.97	0.93	0.96	1.00	0.92	0.80	0.86	1.00	0.84	0.68	0.81
Noman *et al.*[17]	1.00	0.45	0.39	0.27	1.00	0.73	0.57	0.44	0.92	0.75	0.57	0.39	0.89	0.79	0.61	0.38
Kimura [48]	1.00	0.84	0.68	0.58	-	-	-	-	-	-	-	-	-	-	-	-
S-Tree [39]	1.00	1.00	1.00	1.00	-	-	-	-	-	-	-	-	-	-	-	-
Hasan *et al.*[49]	1.00	0.45	0.39	0.27	1.00	0.73	0.57	0.44	1.00	0.68	0.52	0.40	-	-	-	-
DPSO -L1 [50]	1.00	1.00	1.00	1.00	1.00	0.81	0.65	0.54	-	-	-	-	0.89	0.75	0.55	0.32
LTV [51]	1.00	0.73	0.80	0.72	1.00	0.70	0.75	0.66	0.90	0.60	0.69	0.51	-	-	-	-
BANJO [43]	0.42	0.77	0.63	0.50	0.42	0.70	0.56	0.48	0.42	0.70	0.56	0.48	0.33	0.70	0.50	0.40
BITGRN2 [42]	0.92	0.77	0.79	0.85	-	-	-	-	-	-	-	-	-	-	-	-

##### B. Networks with delay (Conf-2 and Conf-3)

**Figure 4:**
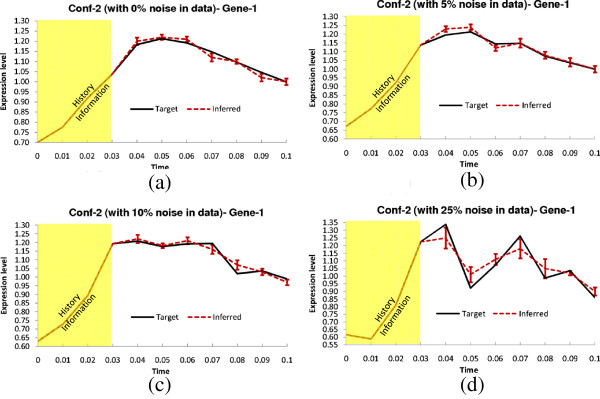
**Dynamics for Gene-1 of Conf-2.** Solid lines and dotted lines indicate respectively target and inferred (by TDSS) time-expressions in **(a)** Noise free data **(b)** 5% Noise in data **(c)** 10% Noise in data **(d)**25% Noise in data. The yellow shaded region indicates the history information and the error bars indicate 95% confidence interval.

**Figure 5:**
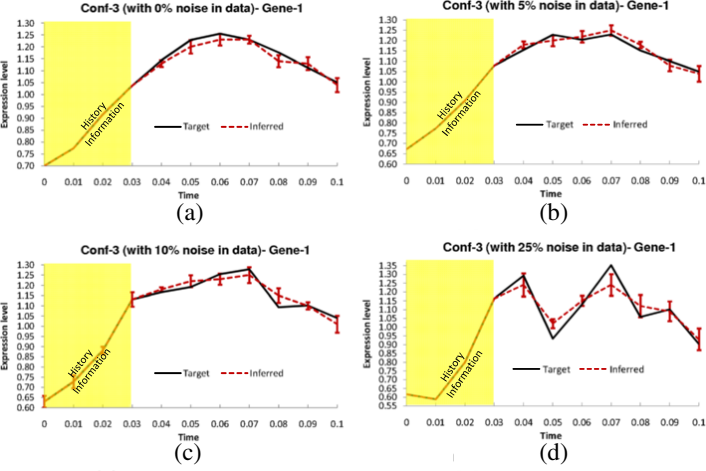
**Dynamics for Gene-1 of Conf-3.** Solid lines and dotted lines indicate respectively target and inferred (by TDSS) time-expressions in **(a)** Noise free data **(b)** 5% Noise in data **(c)** 10% Noise in data **(d)**25% Noise in data. The yellow shaded region indicates the history information and the error bars indicate 95% confidence interval.

The delay parameters (i.e., *τ*^*g*^ and *τ*^*h*^) for the two time-delayed network configurations (Conf-2 and Conf-3) are shown in Table 2. For Conf-2, 5 out of 13 arcs were randomly chosen and applied a delay of 1TS. For Conf-3, we randomly selected six arcs and assigned random fractional delays (in step of 0.1TS) within [0, 3] TS. The delays used in the latter configuration (Conf-3) are designed to validate the method on networks having fractional delays. Similar to the no-delay configuration, we have tested the performance of TDSS with noise-free data and also with three different levels of noise. The four performance metrics for TDSS along with three other existing methods are shown in Table 4. While the previous S-System based methods ALG [34] and REGARD [19], and the BN based approach BANJO [43] considered all inferred edges as instantaneous, TDSS was able to not only infer and segregate these interactions correctly as instantaneous or time-delayed, but the delays were found to be in close agreement to the actual values. The slight deviations between the predicted delays and their actual values might be due to effect of decoupling S-System equations, and also due to the approximation of data with linear spline interpolation. Since the minimum delay possible is 0.1TS, it is reasonable to accept a deviation of ±0.1TS from its predicted value. From this point of view, an instantaneous interaction in original network appearing with a delay of 0.1TS should be deemed accurate. We observe that, in the presence of noise, all the performance measures of TDSS clearly outperform ALG [34], REGARD [19], and BANJO [43]. The time-responses for gene-1 in all conditions are shown in Figure 4(a-d) and Figures 5(a-d) for Conf-2 and Conf-3, respectively. From the four performance metrics and time-responses for TDSS, it is apparent that the proposed method is very efficient in detecting both instantaneous and delayed regulations, as well as accurately capturing gene expression dynamics. In Sec. 3 of the supplementary document (Additional file 1), the time responses for one more gene and the best case parameter sets inferred by TDSS on noise-free data for both Conf-2 and Conf-3 are shown. The inferred networks for Conf-2 and Conf-3 from noise free data (best case) are shown in Figure 2(e) and 2(f), respectively.

**Table 4:** Experimental results on Conf-2 and Conf-3 (5-gene synthetic network)

	**Conf-2 (Delayed network)**															
	**0% Noise**				**5% Noise**				**10% Noise**				**25% Noise**			
	***S***_***n***_	***S***_***p***_	***P***_***r***_	***F***	***S***_***n***_	***S***_***p***_	***P***_***r***_	***F***	***S***_***n***_	***S***_***p***_	***P***_***r***_	***F***	***S***_***n***_	***S***_***p***_	***P***_***r***_	***F***
TDSS (Best)	1.00	1.00	1.00	1.00	1.00	1.00	1.00	1.00	1.00	0.92	0.81	0.90	1.00	0.84	0.68	0.82
TDSS	1.00 ±	1.00 ±	1.00 ±	1.00 ±	1.00 ±	0.98 ±	0.96 ±	0.98 ±	0.98 ±	0.90 ±	0.77 ±	0.87 ±	0.97 ±	0.83 ±	0.67 ±	0.80 ±
(Average ±Std)	0.00	0.00	0.00	0.00	0.00	0.02	0.06	0.03	0.03	0.02	0.04	0.03	0.04	0.02	0.03	0.03
ALG [34]	0.92	0.78	0.60	0.72	0.92	0.78	0.60	0.73	0.77	0.78	0.56	0.65	0.77	0.78	0.56	0.65
REGARD [19]	0.92	0.95	0.86	0.89	0.92	0.95	0.86	0.89	0.85	0.87	0.69	0.76	0.77	084	0.63	0.70
BANJO [43]	0.42	0.77	0.63	0.50	0.42	0.70	0.56	0.48	0.42	0.70	0.56	0.48	0.33	0.70	0.50	0.40
	**Conf-3 (Delayed network)**															
	**0% Noise**				**5% Noise**				**10% Noise**				**25% Noise**			
	***S***_***n***_	***S***_***p***_	***P***_***r***_	***F***	***S***_***n***_	***S***_***p***_	***P***_***r***_	***F***	***S***_***n***_	***S***_***p***_	***P***_***r***_	***F***	***S***_***n***_	***S***_***p***_	***P***_***r***_	***F***
TDSS (Best)	1.00	1.00	1.00	1.00	1.00	1.00	1.00	1.00	1.00	0.95	0.87	0.93	0.92	0.84	0.67	0.78
TDSS	1.00 ±	1.00 ±	1.00 ±	1.00 ±	0.99 ±	0.98 ±	0.94 ±	0.96 ±	0.99 ±	0.92 ±	0.83 ±	0.89 ±	0.88 ±	0.80 ±	0.60 ±	0.71 ±
(Average ±Std)	0.00	0.00	0.00	0.00	0.03	0.02	0.06	0.03	0.03	0.06	0.11	0.08	0.04	0.02	0.02	0.02
ALG [34]	0.85	0.87	0.69	0.76	0.77	0.79	0.56	0.65	0.77	0.78	0.50	0.60	0.70	0.73	0.48	0.56
REGARD [19]	0.85	0.95	0.85	0.85	0.77	0.92	0.77	0.77	0.77	0.81	0.67	0.71	0.77	0.73	0.50	0.60
BANJO [43]	0.42	0.70	0.56	0.48	0.42	0.62	0.50	0.46	0.30	0.62	0.44	0.39	0.25	0.54	0.33	0.29

#### ***Medium scale synthetic network***

**Figure 6:**
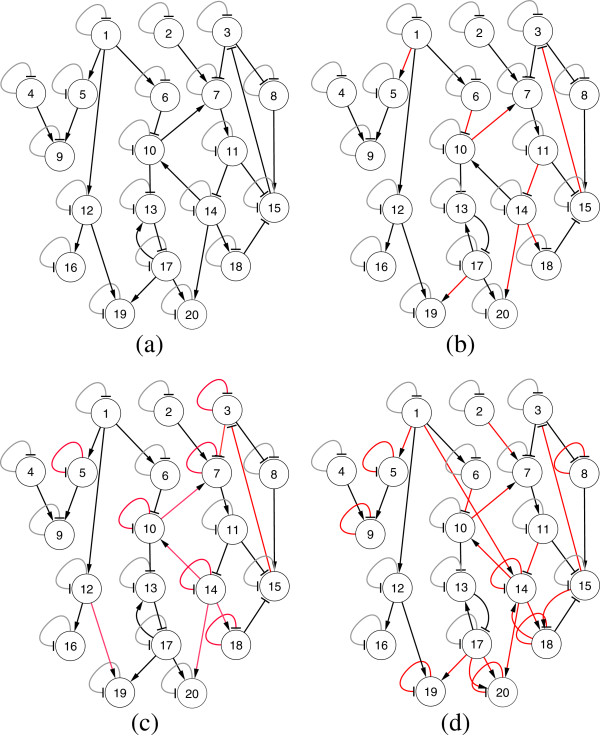
**20-gene networks. (a) **Conf-4 (Target) **(b)**Conf-5 (Target) **(c)** Conf-4 (Inferred) **(d)** Conf-5 (Inferred). Arrow ended black lines and block ended gray lines indicate instantaneous activation and suppression, respectively, while red lines indicate time-delayed regulations.

We now study a medium scale 20-gene synthetic network investigated in [34] and [24]. This network has 20 self-inhibitions in the degradation phase and 26 regulations in the production phase. The target kinetic order and rate constant parameters are shown in Table 5. We investigate two different configurations of this network: one with instantaneous regulations only (Conf-4), and another with both instantaneous and time-delayed interactions (Conf-5). The two different configurations are shown in Figure 6, with their respective delay parameters shown in Table 6.

**Table 5:** **S-System parameters for the 20-gene synthetic network **[34]

*α*_*i*_,*β*_*i*_	10.0
*g*_*i*,*j*_	*g*_**3,15**_ = -0.7,*g*_**5,1**_ = 1.0,*g*_**6,1**_ = 2.0,*g*_**7,2**_ = 1.2,*g*_**7,3**_ = -0.8,*g*_**7,10**_ = 1.6,*g*_**8,3**_ = -0.6,*g*_**9,4**_ = 0.5,*g*_**9,5**_ = 0.7,*g*_**10,6**_ = -0.3,*g*_**10,14**_ = 0.9,*g*_**11,7**_ = 0.5,*g*_**12,1**_ = 1.0,*g*_**13,10**_ = -0.4,*g*_**13,17**_ = 1.3,*g*_**14,11**_ = -0.4,*g*_**15,8**_ = 0.5,*g*_**15,11**_ = -1.0,*g*_**15,18**_ = -0.9,*g*_**16,12**_ = 2.0,*g*_**17,13**_ = -0.5,*g*_**18,14**_ = 1.2,*g*_**19,12**_ = 1.4,*g*_**19,17**_ = 0.6,*g*_**20,14**_ = 1.0,*g*_**20,17**_ = 1.5, other *g*_*i*,*j*_** = 0**
*h*_*i*,*j*_	1.0 if (*i*=*j*), 0.0 otherwise, ∀*i*,*j* = 1,2…,20

**Table 6:** Two different delay configurations of the 20-gene synthetic network

Configuration 4	$\tau_{i,j}^{g}=\tau_{i,j}^{h}=0$ (Non-delayed network)
(Conf-4)	∀*i*,*j* = 1,…,20
Configuration 5	$\tau_{3,15}^{g}=1.1,\tau_{5,1}^{g}=1.3,\tau_{7,10}^{g}=1.6,\tau_{10,6}^{g}=2.1,\tau_{14,11}^{g}=1.5,\tau_{18,14}^{g}=1.9,\tau_{19,17}^{g}=0.6$ ,
(Conf-5)	$\tau_{20,14}^{g}=1.0$ , and remaining $\tau_{i,j}^{g}=\tau_{i,j}^{h}=0,\forall i,j=1,2\ldots ,20$

##### A. Network with no-delay (Conf-4)

**Figure 7:**
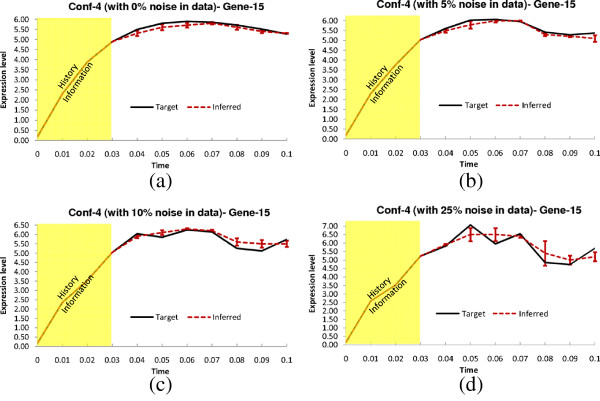
**Dynamics for Gene-15 of Conf-4.** Solid lines and dotted lines indicate respectively target and inferred (by TDSS) time-expressions in **(a)** Noise free data **(b)** 5% Noise in data **(c)** 10% Noise in data **(d)** 25% Noise in data. The yellow region indicates the history information and the error bars indicate 95% confidence interval.

From Table 7, it can be observed that, for noise-free and 5%-noise in data, the proposed technique successfully inferred all the regulations. While TDSS missed a few regulations with higher levels of noise, all the performance measures are observed to be the best among all considered methods. We show the actual and inferred expression dynamics for gene-15, which exhibits high variation throughout the time course, in Figure 7(a-d) for all the four noise levels. The time responses for another selected gene (gene-18) are shown in Sec. 3 of the supplementary document (Additional file 1). The inferred parameter set for the selective genes on this configuration (for noise free data) are also listed in Sec. 3 in the supplementary document (Additional file 1).

**Table 7:** Experimental results on the 20-gene network

	**Conf-4 (No-delay network)**															
	**0% Noise**				**5% Noise**				**10% Noise**				**25% Noise**			
	***S***_***n***_	***S***_***p***_	***P***_***r***_	***F***	***S***_***n***_	***S***_***p***_	***P***_***r***_	***F***	***S***_***n***_	***S***_***p***_	***P***_***r***_	***F***	***S***_***n***_	***S***_***p***_	***P***_***r***_	***F***
TDSS (Best)	1.00	1.00	1.00	1.00	1.00	1.00	1.00	1.00	0.98	0.91	0.60	0.74	0.91	0.90	0.53	0.67
TDSS	0.98 ±	0.97 ±	0.81 ±	0.88 ±	0.96 ±	0.90 ±	0.60 ±	0.72 ±	0.96 ±	0.90 ±	0.56 ±	0.71 ±	0.90 ±	0.87 ±	0.47 ±	0.62 ±
(Average ±Std)	0.01	0.03	0.13	0.01	0.03	0.06	0.23	0.19	0.01	0.01	0.02	0.02	0.01	0.01	0.03	0.03
ALG [34]	0.98	0.85	0.47	0.63	0.98	0.84	0.44	0.61	0.85	0.90	0.54	0.69	0.87	0.86	0.44	0.58
REGARD [19]	0.98	0.90	0.56	0.71	0.98	0.87	0.49	0.65	0.96	0.86	0.56	0.70	0.89	0.87	0.47	0.61
DPSO-L1^*^[50]	0.93	1.00	1.00	0.90	-	-	-	-	0.71	1.00	1.00	0.61	-	-	-	-
BANJO [43]	0.67	0.85	0.35	0.46	0.62	0.79	0.27	0.38	0.56	0.75	0.22	0.31	0.44	0.70	0.16	0.24
BITGRN2 [42]	0.70	0.85	0.40	0.50	0.60	0.85	0.38	0.45	0.55	0.84	0.30	0.40	-	-	-	-
	**Conf-5(Delayed network)**															
	**0% Noise**				**5% Noise**				**10% Noise**				**25% Noise**			
	***S***_***n***_	***S***_***p***_	***P***_***r***_	***F***	***S***_***n***_	***S***_***p***_	***P***_***r***_	***F***	***S***_***n***_	***S***_***p***_	***P***_***r***_	***F***	***S***_***n***_	***S***_***p***_	***P***_***r***_	***F***
TDSS (Best)	1.00	0.96	0.73	0.84	0.98	0.96	0.73	0.89	0.96	0.90	0.56	0.67	0.93	0.89	0.51	0.66
TDSS	0.96 ±	0.95 ±	0.72 ±	0.82 ±	0.96 ±	0.92 ±	0.61 ±	0.75 ±	0.95 ±	0.88 ±	0.51 ±	0.66 ±	0.92 ±	0.89 ±	0.50 ±	0.65 ±
(Average ±Std)	0.02	0.01	0.07	0.04	0.02	0.01	0.01	0.01	0.01	0.01	0.03	0.03	0.01	0.01	0.01	0.01
ALG [34]	0.89	0.85	0.43	0.58	0.87	0.87	0.46	0.60	0.83	0.81	0.45	0.60	0.78	0.79	0.37	0.51
REGARD [19]	0.91	0.88	0.48	0.63	0.91	0.87	0.48	0.63	0.87	0.80	0.47	0.61	0.77	0.75	0.41	0.57
BANJO [43]	0.60	0.79	0.26	0.36	0.56	0.73	0.21	0.30	0.49	0.70	0.17	0.26	0.44	0.69	0.15	0.23

^*^Calculated from average result reported in the paper.

##### B. Network with delay (Conf-5)

**Figure 8:**
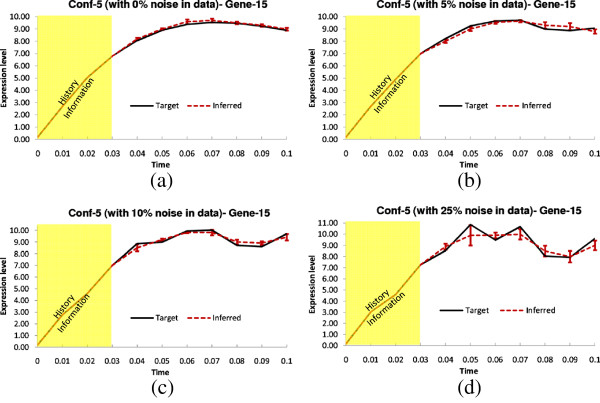
**Dynamics for Gene-15 of Conf-5.** Solid lines and dotted lines indicate respectively target and inferred (by TDSS) time-expressions in **(a)** Noise free data **(b)** 5% Noise in data **(c)** 10% Noise in data **(d)**25% Noise in data. The yellow region indicates the history information and the error bars indicate 95% confidence interval.

This configuration is generated in a similar manner to Conf-3 of the 5-gene synthetic network, with 8 randomly assigned delayed interactions. The experimental results for this configuration are shown in Table 7. The three existing methods ALG [19,34], and BANJO [43] do not handle time-delayed regulations, hence considered all the inferred regulations as instantaneous. Due to the presence of time-delayed regulations, all existing methods missed various true regulations (both instantaneous and time-delayed). On the other hand, for noise-free data, TDSS has successfully recovered the true regulations of the target network. In presence of noise, TDSS performance gradually degraded, but still significantly outperformed the three other techniques. Figure 8(a-d) show the target and inferred expression dynamics for gene-15. The time responses for another gene (gene-8) are shown in Sec. 3 of the supplementary document (Additional file 1). The inferred parameter set for the selective genes on Conf-5 in noise free condition are also listed in Sec. 3 in the supplementary document (Additional file 1).

### Real-life biological networks

#### ***The IRMA network***

**Figure 9:**
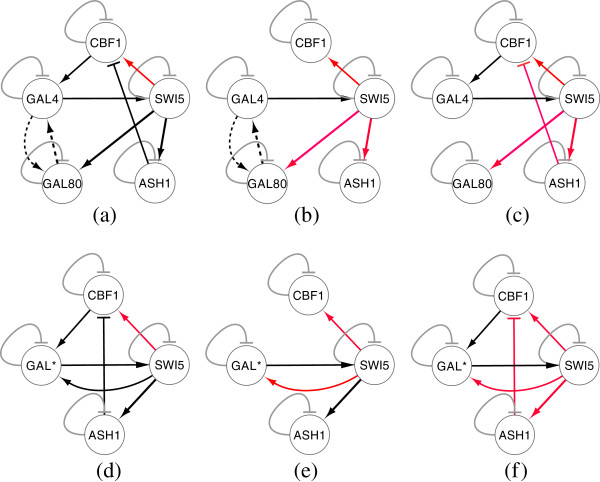
**IRMA networks(original). (a)** Target **(b)** Inferred from ON dataset and **(c)** inferred from OFF dataset. IRMA networks(simplified): **(d)** Target **(e)** Inferred from ON dataset and **(f)** inferred from OFF dataset. Node *GAL*^**∗**^ represents *GAL*4 and *GAL*80. Arrow ended black lines and block ended gray lines indicate instantaneous activation and suppression, respectively, while red lines indicate time-delayed regulations. Dotted lines in **(a)** and **(b)** indicate protein-protein interactions.

We now consider the well-studied IRMA network, a real-life in-vivo synthetic network constructed within the *Saccharomycescerevisiae* yeast [12]. This is a small scale network composed of five genes (*CBF1*, *GAL4*, *SWI5*, *GAL80*, *ASH1*) having a total of 8 regulations. Two gene expression data sets were collected from [12]: the ON data set corresponds to the shifting of the growth medium from glucose to galactose, while the OFF data set corresponds to shifting from galactose to glucose. In the ON (OFF) dataset, there are 16(21) time-samples which were evenly sampled every 20(10) minutes respectively. For the sake of uniformity with the OFF dataset, we have intrapolated (using linear spline interpolation) an additional data point between every two samples in the ON dataset to make it a uniform 10-minute sampled data set. Moreover, as in [39], we also consider the presence of self-inhibition in degradation phase for each genes (i.e., *h*_*i*,*i*_ > 0). It is noted that the mutual interactions between *GAL4* and *GAL80* are protein-protein interactions, which, in principle, are not reflected in gene expression data. Thus, Cantone *et al*. [12] also considered a ‘simplified’ network by combining *GAL4* and *GAL80*, and ignoring their mutual regulations. The IRMA original and simplified networks are shown in Figure 9(a) and Figure 9(d), respectively.

**Figure 10:**
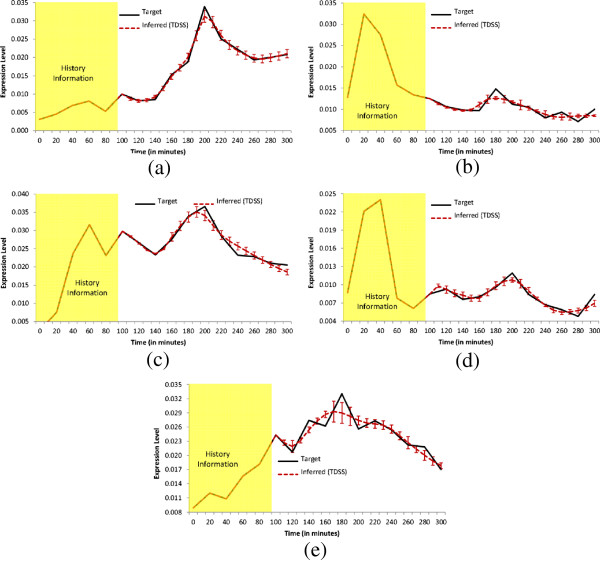
**Dynamics for 5 genes in IRMA ON network, solid lines and dashed lines indicate target and inferred dynamics, respectively and the error bars indicate 95% confidence interval. (a)** CBF1 **(b)** GAL4 **(c)** SWI5 **(d)** GAL80 **(e)** ASH1.

The experimental results for the IRMA network are shown in Tables 8 and 9. We note that, for the ON data set, TDSS was successful in inferring a higher number of true regulations (11 out of 13) than any other methods reported in this paper. As a result, the sensitivity is highest amongst all the methods (*S*_*n*_ = 0.85), while the other performance metrics *S*_*p*_ and *P*_*r*_ are very close to the best values. More importantly, the F-score (*F*), which is the harmonic mean of precision and sensitivity, is relatively high for TDSS (second best). While considering the simplified network, although the performance metrics of TDSS are not the best among the methods, they are still very competitive and close to the best values. The inferred network with only true regulations for both original and simplified structure are shown in Figure 9(b) and Figure 9(c), respectively. On the OFF data set, TDSS also exhibits good performance, with the best *S*_*n*_ and *F* among all considered methods. Further, on the simplified network, all the four performance measures are found best for the TDSS. The TDSS time responses for all genes on the ON data sets in Figure 10 clearly indicate that the inferred gene expressions are very close to the corresponding targets.

**Table 8:** Experimental results for IRMA network, reconstructed from ON dataset

	**Original network**				**Simplified network**			
**Methods**	***S***_***n***_	***S***_***p***_	***P***_***r***_	***F***	***S***_***n***_	***S***_***p***_	***P***_***r***_	***F***
TDSS (Best)	**0.85**	0.86	0.69	0.76	**0.80**	0.92	0.80	**0.80**
TDSS	0.80 ±	0.84 ±	0.64 ±	0.71 ±	0.76 ±	0.89 ±	0.75 ±	0.75 ±
(Avg ±StDev)	0.04	0.02	0.04	0.04	0.05	0.02	0.04	0.03
ALG [16]	0.77	0.27	0.27	0.40	**0.80**	0.42	0.36	0.50
REGARD [19]	0.69	0.83	0.60	0.64	0.70	0.75	0.54	0.61
BITGRN2 [24,42]	0.63	**1.00**	**1.00**	**0.77**	0.67	**1.00**	**1.00**	**0.80**
TDARACNE [44]	0.63	0.88	0.71	0.67	0.67	0.90	0.80	0.73
ARACNE [7]	0.60	–	0.50	0.54	0.33	–	0.25	0.28
NIR & TSNI [45]	0.50	0.94	0.80	0.63	0.50	–	0.50	0.50
BANJO [43]	0.24	0.76	0.33	0.29	0.50	0.70	0.50	0.50

**Table 9:** Experimental results for IRMA network, reconstructed from OFF dataset

	**Original network**				**Simplified network**			
**Methods**	*S*_*n*_	*S*_*p*_	*P*_*r*_	*F*	*S*_*n*_	*S*_*p*_	*P*_*r*_	*F*
TDSS (Best)	**0.85**	0.81	0.65	**0.73**	**1.00**	**0.92**	**0.83**	**0.91**
TDSS	0.80 ±	0.83 ±	0.63 ±	0.70 ±	0.90 ±	0.87 ±	0.75 ±	0.81 ±
(Avg ±StDev)	0.04	0.02	0.03	0.02	0.07	0.03	0.06	0.06
ALG [16]	0.76	0.56	0.38	0.57	0.80	0.75	0.57	0.67
REGARD [19]	0.77	0.76	0.53	0.63	0.80	0.79	0.62	0.70
BITGRN2 [24,42]	0.50	**0.94**	**0.80**	0.62	0.50	0.90	0.75	0.60
TDARACNE [44]	0.60	-	0.37	0.46	0.75	-	0.50	0.60
ARACNE [7]	0.33	-	0.25	0.28	0.60	-	0.50	0.54
NIR & TSNI [45]	0.38	0.88	0.60	0.47	0.50	0.90	0.75	0.60
BANJO [43]	0.38	0.88	0.60	0.46	0.33	0.90	0.67	0.44

Additionally, we highlight here an interesting biological finding made during the computational analysis. In particular, the proposed S-System model was successful in uncovering the important time-delay interaction in the IRMA network for the activation of *CBF1* from *SWI5*. More specifically, from observations during the in-vivo experiment, this regulation was experimentally characterized as a time-delayed interaction of 100 minutes [12]. The proposed S-System model is the first-ever method that discovered, not only this time-delayed nature of the interaction, but also the accurate time-delay value (in minutes). In particular, for the IRMA-ON data set, the *SWI5* →*CBF1* interaction was inferred as a 94-minute delayed regulation. The same regulation was also successfully inferred as a delayed regulation of 92 minutes while reconstruction was performed with the IRMA-OFF dataset. Both the delay values are very close to the original time delay value of 100 minutes as reported in [12]. All the inferred true regulations along with corresponding time-lags are listed in Table 10. Indeed, we believe that this interesting finding is made possible due to the novel features present in the proposed TDSS model.

**Table 10:** **Regulations within the IRMA network inferred by TDSS with corresponding *τ * values**

**Inferred**	**IRMA-ON**		**IRMA-OFF**	
**regulations**			**lag( *τ *) values**	
**by TDSS**	**Time-stamps**	**Minutes**	**Time-stamps**	**Minutes**
*SWI*5 → *CBF*1	9.4	94	9.2	92
*SWI*5 → *GAL*80	2.3	23	1.7	17
*SWI*5 → *ASH*1	1.8	18	1.0	10
*GAL*4 → *SWI*5	0.0	0	0.0	0
*GAL*4 → *GAL*80	0.0	0	-	-
*GAL*80 → *GAL*4	0.0	0	-	-
*ASH*1 ⊣ *CBF*1	-	-	0.4	4
*CBF*1 → *GAL*4	-	-	0.0	0

#### ***The SOS DNA repair network in Escherichia coli***

Next, we consider the well-studied SOS DNA repair network within *Escherichiacoli* (*E. coli*). While the entire DNA repair system of *E.coli* involves more than 100 genes [39,47], only its 30 genes contribute towards key regulations at the transcription level. We make use of the expression data set collected by Ronen *et al.*[52], which contains information about 8 genes namely *uvrD*, *lexA*, *umuD*, *recA*, *uvrA*, *uvrY*, *ruvA*, and *polB*. The data sets are obtained from four different experiments under various UV light conditions, with the gene expression levels being measured at 50 instants evenly spaced at a 6-minute interval. Following [34,40,46], we normalize the input data by dividing the expression profile of each gene by its maximum value. Historically, there were two versions of this SOS network in the literature, one involving 6 genes (*uvrD*, *lexA*, *umuD*, *recA*, *uvrA* and *polB*) [19,39,46], and another involving all the 8 genes [23,24,43,47], both inferred from Ronen *et al.*’s expression data [52]. Herein, we study both the networks.

As the exact ground truth for this network is not precisely known, it is not possible to calculate the four performance metrics, i.e., sensitivity, specificity, precision and F-score. However, from the functional description of each gene in the original paper [52], it is generally recognized that suppressions of all genes from *lexA* and activation to *lexA* from *recA* are considered as true regulations. On the 6-gene SOS network, TDSS successfully inferred 6 out of these 7 known regulations, missing only the activation *recA* →*lexA*. Authors in [19,39,46] also considered this 6-gene SOS network and successfully inferred 5, 6, and 5 true regulations, respectively, as detailed in Table 9. The method of Kimura et al. [53] inferred all the 7 known regulations, however, with all incorrect regulatory signs. For the 8-gene SOS network, ALG [34] and several BN based approaches, namely BANJO [43], Perrin et al. [47], GlobalMIT [23], and BITGRN2 [24] respectively inferred 6, 2, 4, 5, 4 true regulations. The proposed TDSS successfully inferred 7 known regulations, as detailed in Table 11. For the 8-gene SOS network, all the methods considered in this comparison, including TDSS, failed to infer the regulation *lexA* →*ruvA*.

**Table 11:** **“True”+“Novel” interactions of *E. coli * network inferred by TDSS and other state-of-the-art methods**

	**Considering 6-gene subnetwork**					**Considering 8-gene subnetwork**					
**True**	**Proposed**	**REGARD**	**S-Tree**	**NGnet**	**Kimura**	**Proposed**	**ALG**	**Perrin**	**BANJO**	**GlobalMIT**	**BITGRN2**
**positives**	**(TDSS)**	[19]	[39]	[46]	***et al *** [53]	**(TDSS)**	[34]	***et al *** [47]	[43]	[23]	[24]
*lexA*⊣*recA*	*√*	*√*	*√*	*√*	⋆	*√*	*√*	*√*		*√*	*√*
*lexA*⊣*lexA*	*√*	*√*	*√*	*√*	⋆	*√*	*√*	*√*			
*lexA*⊣*umuD*	*√*	*√*	*√*	*√*	⋆	*√*	*√*			*√*	*√*
*lexA*⊣*uvrD*	*√*	*√*		*√*	⋆	*√*	*√*				*√*
*lexA*⊣*uvrA*	*√*	*√*	*√*		⋆	*√*	*√*	*√*	*√*	*√*	*√*
*lexA*⊣*polB*	*√*		*√*	*√*	⋆	*√*				*√*	
*recA*→*lexA*			*√*	⋆	⋆		*√*	*√*	*√*		
*lexA*⊣*uvrY*						*√*				*√*	
*lexA*⊣*ruvA*											
**Total TP inferred**	6	5	6	5	0	7	6	4	2	5	4
**Novel Interactions**											
*umuD*→*lexA*	*√*	*√*			⋆	*√*	*√*	*√*		*√*	
*uvrA*⊣*lexA*	*√*	*√*		⋆	*√*	*√*		*√*			
*uvrA*⊣*recA*			*√*	⋆	⋆			*√*	*√*	*√*	
**Total Novel Interactions**	2	2	1	0	1	2	1	3	1	2	0

⋆: Inferred with incorrect regulatory sign.

It should be noted that, other than the known regulations reported in Table 11, considered as true positives, the proposed TDSS also inferred some unknown regulations. These can be either novel regulatory interactions, or false positive findings. These interactions are shown as “Novel Interactions” in Table 11. We refer to the existing state-of-the-art methods where these unknown regulations were justified. For example, the regulation of *lexA* by *umuD* was previously discovered and discussed in [47] and [16]**,**[34]. This regulation was also discovered by two of our previously proposed methods REGARD [19] and GlobalMIT [23]. This regulation is inferred by the proposed TDSS on both the 6-gene and 8-gene networks. Further, the regulation *uvrA* ⊣*lexA*  was also inferred by TDSS for both networks. This interaction was also previously reported in [47] and [53]. Finally, the regulation *uvrA* ⊣*recA* was inferred by 4 existing methods [23]**,**[39]**,**[43]**,**[47], while TDSS did not discover this connection. Historically, all these three novel regulations mentioned in Table 11 were first reported by Perrin *et al.*[47], and later re-discovered by other methods [16]**,**[43]. However, for confirming the biological validity of these interactions, suitable wet-lab experiments are yet to be performed. It is noted that for TDSS and other S-System based methods, self-regulations in either or both the production or degradation phase is normally needed to balance the model. However we clarify that, self-regulations in DE based approaches reflect the self-dependency of a gene expression upon its own value at a previous time point, rather than a physical self-interaction.

**Figure 11:**
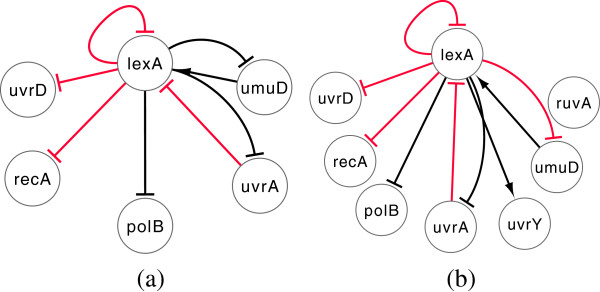
**True regulations inferred by TDSS considering. (a)** 6-gene subnetwork. **(b)** 8-gene subnetwork. Solid black lines and red lines indicate instantaneous and delayed regulations in production phase, respectively.

**Figure 12:**
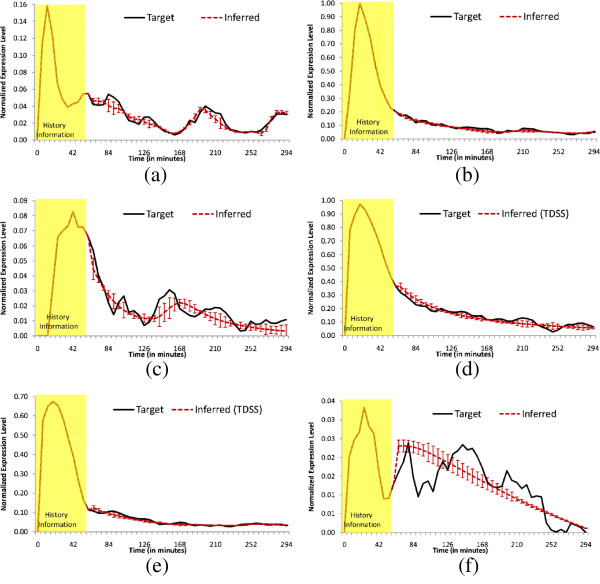
**Dynamics for 6 genes in *E. coli * network, solid lines and dashed lines indicate target and inferred dynamics, respectively and the error bars indicate 95% confidence interval. (a)***uvr*D **(b)***lex*A **(c)***umu*D **(d)***rec*A **(e)***uvr*A **(f)***pol*B.

For the 6-gene (8-gene) SOS network, the proposed TDSS method was successful in inferring 8 (9) “true”+“novel” regulations, including 4 (5) regulations which were reported as time-delayed. These results indicate the presence of possible delayed regulations in the network. All the inferred true regulations are shown in Table 12 with their corresponding time-lags. The true regulations inferred correctly in TDSS for both the sub-networks are shown in Figure 11(a) and Figure 11(b). Further, Figure 12 shows that, despite the inherent noise in real-life data, TDSS time responses for all the 6 genes are very close to the target expression patterns.

**Table 12:** **Regulations of *E. coli * SOS network inferred by TDSS with corresponding *τ * values**

**Inferred**	**6-gene network**		**8-gene network**	
**regulations**	**Lag( *τ *) values**		**Lag( *τ *) values**	
**by TDSS**	**Time-stamps**	**Minutes**	**Time-stamps**	**Minutes**
*lexA*⊣*uvrD*	1.8	10.8	1.9	11.4
*lexA*⊣*lexA*	0.7	4.2	0.6	3.6
*lexA*⊣*umuD*	0.0	0.0	0.1	0.6
*lexA*⊣*recA*	2.1	12.6	2.3	13.8
*lexA*⊣*uvrA*	0.0	0.0	0.0	0.0
*lexA*⊣*polB*	0.0	0.0	0.0	0.0
*umuD*→*lexA*	0.0	0.0	0.0	0.0
*uvrA*⊣*lexA*	2.1	12.6	1.9	11.4
*lexA*⊣*uvrY*	-	-	0.0	0.0

### Computational efficiency

**Figure 13:**
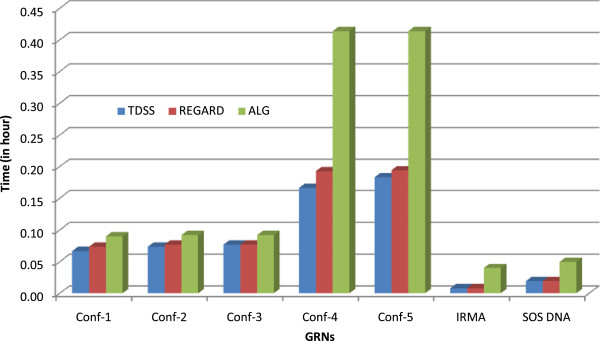
**Run time comparisons of TDSS with two existing methods (ALG **[34]** and REGARD **[19]**).** The Y-axis shows the average computation time, in hours, required to infer a single gene (in decoupled S-System).

**Figure 14:**
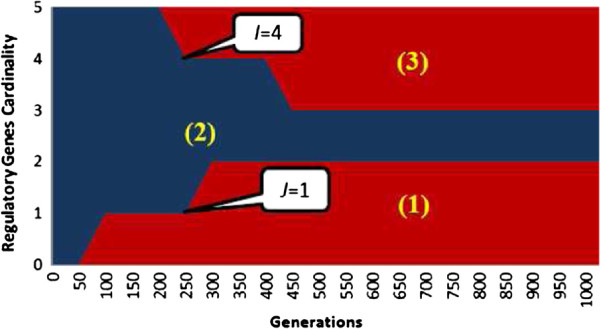
**Effect of *I *and *J *in the optimization. **(1), (2), and (3) respectively indicate the regions where *r*_*i *_is less than *J*, within [*J*,*I*] range, and greater than *I*.

Finally, we consider the issue of computational time. We have compared the timing of TDSS with two other S-System based approaches, namely REGARD [18] and ALG [34]. The average times for these three methods to infer the parameters of a single gene on seven networks considered in this paper are shown in Figure 13. We observe that, despite a significant increase in the number of parameters to model the time delay, TDSS was found to converge much faster than ALG [34], and marginally faster than REGARD [18]. This demonstrates the benefit of dynamically adapting the regulatory genes cardinality (i.e., minimum *J* and maximum *I* in-degrees) as explained in the proposed Methods section. The adaptation of *I* and *J* narrows down the search space significantly and speeds up convergence. In Figure 14, we show the optimization process for gene-1 of Conf-1 (5-gene network) in a particular run.

## Conclusion

Time-delayed regulations are an inherent characteristics of all biological networks. While there have been some recent efforts using Bayesian network (BN) approach to simultaneously model time-delayed and instantaneous interactions, the current state of the art S-System approaches cannot model time-delayed interactions. In this paper, we have proposed a novel method to incorporate time-delayed interactions in the existing S-System modeling approaches for reverse engineering genetic networks. The proposed Time-delayed S-System (TDSS) model is capable of simultaneously representing both instantaneous and time-delayed regulations. Apart from the kinetic order and rate constant parameters as in traditional S-System models, additional parameters for the time delays are necessary for TDSS full description. To make the optimization effective and efficient in the increased parameter space, we proposed a novel objective function based on the sparse and scale-free nature of genetic network. The inference method was also redesigned, based on adaptive systematic adaptation of the max and min in-degrees for gene cardinality, and systematic balancing between time response accuracy and network complexity during the optimization process. The RK4 numerical integration technique has also been suitably adapted for TDSS. Investigations carried on small and medium synthetic networks with various levels of noise, as well as on two real-life genetic networks show that our approach correctly captures the time-delayed interactions and outperforms other existing S-System based methods.

## Competing interests

The authors declare that they have no competing interests.

## Authors’ contributions

ARC, MC and NXV developed the concepts and drafted the manuscript. ARC developed the algorithms and carried out the experiments. MC and NXV suggested the biological data, experiments and provided biological insights on the results. MC provided overall supervision, direction and leadership to the research. All authors read and approved the final manuscript.

## Supplementary Material

Additional file 1 Supplementary Document. Click here for file
