# Supplementary material for: Incorporating time-delays in S-System model for reverse engineering genetic networks
Source: BMC Bioinformatics. 2013 Jun 18;14:196. doi: 10.1186/1471-2105-14-196 (PMC3839642; doi:10.1186/1471-2105-14-196)
Supplement: Additional file 1 — Supplementary Document. [file 1471-2105-14-196-S1.pdf]

Incorporating time-delays in S-System model for reverse engineering genetic networks - Supplementary

Ahsan Raja Chowdhury[1,2*], Madhu Chetty[1,2] and Nguyen Xuan Vinh[1]

[1]Gippsland School of Information Technology, Monash University, Australia
[2]National Information and Communication Technology Australia (NICTA)

Email: [1]{ahsan.chowdhury, madhu.chetty, vinh.nguyen}@monash.edu; [2]{ahsan.chowdhury, madhu.chetty}@nicta.com.au;

[*]Corresponding author

Contents

1 Additional background information **2**
1.1 S-System model . . . 2
1.2 Model evaluation criteria . . . 2
1.3 Parameter learning using evolutionary algorithm . . . 4

2 The method: additional information **4**
2.1 Time delayed S-System (TDSS): The model . . . 4
2.2 Modified Pearson correlation coefficient technique . . . 5
2.3 Modified numerical integration for TDSS . . . 6
2.4 Reverse engineering GRN with TDSS . . . 6
2.4.1 Initial population generation with delay . . . 6
2.4.2 New fitness function . . . 9
2.4.3 Adaptive regulatory genes cardinality . . . 10
2.4.4 Hill climbing local search for delay parameters . . . 11
2.4.5 Multistage refinement algorithm . . . 11

3 Additional experimental results and discussions **12**

Abstract

In this supplementary document, we give additional details in support of the results presented in the main paper. Section 1 offers background information while Section 2 gives more information about our proposed method. Supplementary experimental results, along with tables and graphs, are presented in Section 3.

1 Additional background information

1.1 S-System model

To solve the ODEs of Eqn. (1) in original document, the S-System based models [1–5] used the Runge-Kutta 4th order (RK4) equations. The RK4, an old but efficient technique to perform the numerical integration, uses the tth expression level values for all N genes to generate the value for the $(t+1)^{th}$ level of the gene-i. The RK4 is invoked to approximate the values in between these two timestamps (TSs) (we use the terms timestamp, time-sample, time-interval interchangeably through out this document). While generating the data for the gene-i in the $(t+1)^{th}$ timestamp, RK4 calculates the four slope values from the tth level and calculate the final value with an averaging function, as shown in Figure 1. According to the figure, to generate the $(t+1)^{th}$ level data for gene-i, RK4 includes all the data points of tth level. Hence, implicitly, the S-System modeling implements instantaneous interactions only.

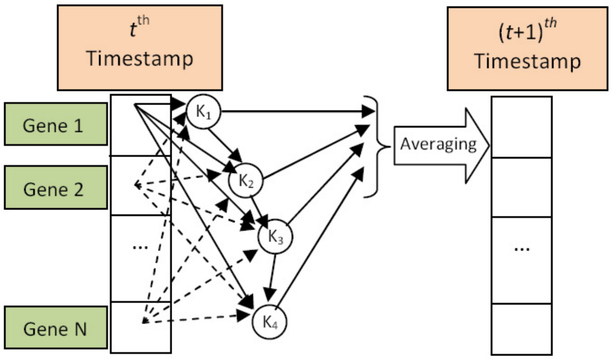

Figure 1: Numerical Integration of RK4 to solve the traditional canonical form of S-System equation. $(t+1)^{th}$ timestamp value for the gene-i is calculated from the data of tth timestamp.

Figure 1 shows the scenario for canonical S-System modeling which requires enormous computation time to converge properly. However, decoupled S-System, can infer the parameters of the target network much quickly, albeit with some approximation, compared to the canonical S-System model. Due to higher number of parameters (number of parameters in canonical S-System is equal to N times number of parameters in decoupled S-System), the search space becomes very vast, which can cause the search process to be trapped in any of the local minima. On the other hand, the decoupled S-System, represented by Eqn. (2) of the original document, requires fewer number of parameters to learn than the canonical S-System and allows converging the optimization rapidly. However, the decoupled form requires approximation of intermediate values for four slopes in the numerical integration of RK4, which are directly calculated in canonical form. As a result, optimization may end up with suboptimal values of the parameters due to this approximations, and may fail to infer the target network with 100% accuracy.

1.2 Model evaluation criteria

Fitness functions play an important role in the evolutionary computations. The performance of the SRE based fitness function (Eqn. (3) of original document) was moderate while addition of model complexity term exhibits even better performance. Due to the inherent sparsity in biological networks, the inference algorithms are often designed to deal with sparse solutions. In this regard, Kimura et al. [2] added a penalty term based on the maximum in-degree I. It was considered that out of $2N$ kinetic parameter values of g and h, only I non-zero values are expected (for each gene) within the g and h vectors, thus leaving the other $2N$-I values to be zero. If any of these $2N$-I elements acquire a non-zero value, that solution is penalized in

the following manner:

$$RSRE_1 = \sum_{t=1}^{T} \left(\frac{X_i^{cal}(t) - X_i^{exp}(t)}{X_i^{exp}(t)} \right)^2 + c \sum_{j=1}^{N-I} (|G_{ij}| + |H_{ij}|) \tag{1}$$

Here, G_{ij} and H_{ij} are populated by sorting the absolute values of g_{ij} and h_{ij} respectively in non-decreasing order of their absolute values. Remaining other terms are defined in Section 2 of the original paper. Later, this Eqn. (1) has been modified by Noman et al.in [6] by combining both the kinetic parameters (Eqn. (4) of the original document).

There also exists other fitness functions based on information criteria, such as AIC, BIC, HQ, GCV etc. Akaikes Information Criteria (AIC) [7] is the most commonly used in statistical modeling to show the discrepancy between the target and estimated models. Let, $\epsilon_i(t)$ be the difference between $X_i^{cal}(t)$ and $X_i^{exp}(t)$ for gene-i. If $\epsilon_i(t)$ is assumed as the normal distribution with mean μ_i=0 and standard deviation σ_i, which are constant over time for gene-i, then log likelihood Λ_i of the expression data for this gene for a set of parameters Ω_i (defined earlier) for gene-i is given by:

$$\Lambda_i(\Omega_i, \sigma_i) = -\frac{1}{\sigma_i^2} \sum_{t=1}^{T} (\epsilon_i(t))^2 - \frac{T}{2} ln(2\pi\sigma_i^2) \tag{2}$$

The maximum likelihood estimate of σ_i^2 is obtained accordingly as follows:

$$\sigma_i^2(max) = \frac{1}{T} \sum_{t=1}^{T} (\epsilon_i(t))^2 \tag{3}$$

Substituting (3) in (2), the log-likelihood of the estimated model is obtained from which, AIC is defined as [7]:

$$AIC = -2\Lambda + 2\Phi \tag{4}$$

Here, Φ is the number of parameters included in the model. This AIC based fitness value has been further modified to Eqn. (5) [6] by combining Eqn. (4) of the original document:

$$f_i^{AIC} = -2\Lambda + 2\Phi + c \sum_{j=1}^{2N-I} (|K_{ij}|) \tag{5}$$

The additional penalty term becomes very useful when number of regulations in the skeletal network is higher than the maximum in-degree. Recently, we have introduced a new fitness function (Eqn. (5) of the original document) based on regulatory genes cardinality that modifies the model complexity part of RSRE based fitness function. The new fitness function introduced the minimum in-degree (J) along with traditional maximum in-degree (I). First, we calculate the number of transcription factors (total regulations, r_i) for gene-i. Thus, with the knowledge of the number of regulations r_i, we can easily determine the penalty term in Eqn. (6) of the original document. Note that, although the term $(\frac{2N}{Z_{Count}})$ encourages an individual to have more non-regulating genes (more zero values), it is also restricting this with a penalty term C_i, which is calculated based on the Max-Min cardinality (Eqn. (6) of the original document). The term $(\frac{2N}{Z_{Count}})$ is considered as N when Z_{Count} calculation ends up with 0 (to avoid divide by zero problem). Although, the penalty graph generated by the model complexity part resembles the property of power-law formalism, penalty term of Eqn. (5) in the original document is the combination of exponential increase and decrease property. Although the initial study on this fitness criteria reported satisfactory result [5], the included penalty function penalized the solution in asymmetric way. Hence, a new penalty function is developed in this research that has a characteristic similar to power-law formalism.

1.3 Parameter learning using evolutionary algorithm

Differential Evolution was first proposed by Storn et al. [8], later modified as Trigonometric Differential Evolution (TDE) [3, 9] by incorporating Trigonometric Mutation Operation [10]. Noman *et al.* initially used (DE) to reverse engineer GRN with the S-System and subsequently applied TDE, the modified version of DE. Conventional Mutual Operation (MO) and Trigonometric Mutual Operation (TMO) can be defined using Eqn. (6) and Eqn. (7) respectively.

$$y_i = x_{r_1} + F_0(x_{r_2} - x_{r_3}) \tag{6}$$

$$y_i = \frac{1}{3}(x_{r_1} + x_{r_2} + x_{r_3}) + (p_{r_2} - p_{r_1})(x_{r_1} - x_{r_2}) + (p_{r_3} - p_{r_2})(x_{r_2} - x_{r_3}) + (p_{r_1} - p_{r_3})(x_{r_3} - x_{r_1}) \tag{7}$$

where,

$$\begin{aligned} p_{r_1} &= |f(x_{r_1})|/p' \\ p_{r_2} &= |f(x_{r_2})|/p' \\ p_{r_3} &= |f(x_{r_3})|/p' \\ p' &= |f(x_{r_1})| + |f(x_{r_2})| + |f(x_{r_3})| \end{aligned} \tag{8}$$

Here, r_1, r_2 and r_3 are positive random numbers not more than total number of individuals (P) such that, $r_1 \neq r_2 \neq r_3 \neq i$, x_k are individuals with $k \in \{r_1, r_2, r_3\}$, F_0 is the TDE parameter (already defined in the experimental section of the original document). Noman et al.used Eqn. (6) and Eqn. (7) based on a probability F_t and $(1 - F_t)$, respectively. They call this mixture of TMO in conventional DE as TDE [11]. This Mutation operation is then followed by crossover and selection operation to form the individuals for next generation. It was found in [3] that, TDE possesses good convergence properties than the well-known DE [8] and also efficient in genetic network inference [9, 11]. As an optimization tool for reverse engineering GRN, both DE and TDE perform better than the other conventional evolutionary computation approaches [4, 6]. The TDE based optimization was also used in our previous optimization [5], which reported promising results for both synthetic and real networks.

Most of the evolutionary approaches for learning the S-System parameters in GRN reconstruction starts by initializing the system parameters randomly for all the individuals. The data is then calculated for every set of parameters (each individuals) to measure the error between experimental and calculated data. A fitness function is invoked in this regard for error calculation. Very few methods rely only on the fitness function to be minimized in subsequent generations, rather local search procedures contribute significantly in faster convergence. Although best fit individuals from current generation survive for next generation, due to the vast search space and possible local minima, the algorithm may end up with sub-optimal solution. This is why the optimization algorithms include a diversification technique, that is triggered when the system is in local minima or no significant improvement occurring for good number of generations. In our previous works, we used "Flip" operation [4, 5], a special type of mutation operation applied over specified number of individuals in certain generation. We also use this Flip operation in our proposed TDSS.

2 The method: additional information

The novel Time Delayed S-System is introduced in Section 3 in the original document. In this section, we present additional information about the TDSS, modified RK4 and reverse engineering using TDSS.

2.1 Time delayed S-System (TDSS): The model

In order to generalize the Time-delayed S-System model (Eqn. (9) of the original document), we have facilitated any interaction to be time-delayed. The traditional S-System model incorporates $2 \times N \times N$ regulations as system parameters and, although impractical, we assume that all $2 \times N \times N$ regulations can be time-delayed. Hence we need a matrix of $2 \times N \times N$ cells to represent the delay parameters. For simplicity,

we consider 2 matrices (τ^g and τ^h), each of having $N \times N$ cells (Eqn. (10) and Eqn. (11) of the original document). For both the matrices, $\{0 \leq \{\tau^g_{i,j}, \tau^h_{i,j}\} \leq \tau_{max}\}$, $\forall_{i,j=1...N}$ and τ_{max} is the maximum allowed delay of the network. Here, $\tau^g_{i,j}$ and $\tau^h_{i,j}$ indicate the delay unit for regulation on gene-i from gene-j in production phase and degradation phase, represented by g and h matrices, respectively. These two matrices are calculated according to the following equations:

$$\tau^g_{i,j} = \begin{cases} D & \text{if } g_{i,j} \text{ is a delayed interaction of } D \text{ time,} \\ & \text{where } 0 < D \leq \tau_{max} \\ 0 & \text{if no interaction from } j \text{ to } i \text{ in the production} \\ & \text{or } g_{i,j} \text{ is an instantaneous interaction} \end{cases} \tag{9}$$

$$\tau^h_{i,j} = \begin{cases} D & \text{if } h_{i,j} \text{ is a delayed interaction of } D \text{ time} \\ & \text{where } 0 < D \leq \tau_{max} \\ 0 & \text{if no interaction from } j \text{ to } i \text{ in the degradation} \\ & \text{or } h_{i,j} \text{ is an instantaneous interaction} \end{cases} \tag{10}$$

The new model requires both τ^g and τ^h to be learned in the optimization. However, we seed different values for D in the initial population based on the knowledge calculated by the statistical measurement Pearson Correlation Coefficient.

2.2 Modified Pearson correlation coefficient technique

We have used Pearson Correlation Coefficient (PCC) technique [12] to initialize the lag values for the interaction based on our improved Pearson Correlation Coefficient (PCC). The conventional PCC is a measure of dependance (linear) between two variables X and Y (genes in our case), and returns a value between +1 and -1 inclusive. If we shift variable Y to s cell(s) and perform the PCC, it will return a value which indicates the mutual interaction of variable X on variable Y with s unit delay. However, due to the non-linear nature of genes' interactions and linearly testing property of the PCC, the precalculation of delay matrices yields the values that have inappropriate meaning in terms of true regulatory network. During optimization, we use a heuristic algorithm to calculate more accurate matrices using PCC and then extract the approximate delay units for each interaction. Lets consider that a GRN of N genes are sampled T times to produce a microarray data. Moreover, there are M datasets, which are produced from M initial conditions for each genes. In between two time samples, we generate 9 more values using Linear Spline Interpolation [13]. At the end, each data set has $10{\times}T$ samples which we will use to generate the delay matrices. After that, we apply the following improvement over standard PCC to obtain the probable delay for the interactions of the given network.

We define the following operation as the standard PCC between G_i and G_j of kth dataset, shifting G_j expression by s cells to the right.

$$\begin{gathered} PCC^k(s)[i,j] \\ s = 0 \ldots 10\tau_{max}, k = 1 \ldots M, i = 1 \ldots N, j = 1 \ldots N \end{gathered} \tag{11}$$

Here, $s = 0$ implies conventional PCC operation, while other values of s define PCC for s unit delay between genes G_i and G_j. We set the maximum value of s to τ_{max} before computing the PCC based on some "prior knowledge" about the network. Every $PCC^k(s)[i,j]$ for a particular s value construct M matrices (for M data sets), each having $N \times N$ cells. According to the following equation, we obtain the average among M absolute values for each interaction:

$$\begin{gathered} PCC^0(s)[i,j] = Average(|PCC^1(s)[i,j]|, |PCC^2(s)[i,j]|, \ldots, |PCC^M(s)[i,j]|) \\ \forall i,j = 1, \ldots, N \\ \forall s = 0, \ldots, \tau_{max} \end{gathered} \tag{12}$$

Then we filter every cells of each matrix based on the threshold ζ (=0.9 for our experiment) and mark the cell by its corresponding s value:

$$
\begin{gathered}
PCC^0(s)[i,j] = \begin{cases} s & \text{if } PCC^0(s)[i,j] > \zeta \\ 0 & \text{otherwise} \end{cases} \\
\forall i,j = 1,\ldots,N \\
\forall s = 0,\ldots,\tau_{max}
\end{gathered} \tag{13}
$$

Finally, we obtain the PCC matrix for a microarray data by taking the maximum lag values among all τ_{max}+1 values for every interaction:

$$
\begin{gathered}
PCC[i,j] = max\{PCC^0(0)[i,j], PCC^0(1)[i,j], \ldots, PCC^0(\tau_{max})[i,j]\} \\
\forall i,j = 1 \ldots N
\end{gathered} \tag{14}
$$

The values of this matrix is considered as the prior knowledge for the delayed regulations and used in the initial population generation.

2.3 Modified numerical integration for TDSS

During the numerical integration, gene expression values for the intervals $[t_0,\, t_{\tau_{max}}]$ requires the values of $[t_{0-\tau_{max}},\, t_0]$ interval, where X_{i,t_0} for all genes ($i = 1,\ldots,N$) are given as initial condition. The expression values of the interval $[t_{0-\tau_{max}},\, t_0]$, known as 'History Information', should be provided as input to the system or handled by some other means. In our proposed framework, we map the region $[t_{0-\tau_{max}},\, t_0]$ to $[t_0,\, t_{0+\tau_{max}}]$ and generate the new 'History Information' with linear spline interpolation technique. The situation is clearly exposed in Figure 2, where the region $[t_{0-\tau_{max}},\, t_0]$ in Figure 2(a) is mapped to $[t_0,\, t_{0+\tau_{max}}]$ in Figure 2(b) and TDSS starts the numerical integration from $t^{th}_{0+\tau_{max}}$ interval.

However, the framework is limited to the situation when τ_{max} is an integer value and the step size (h) is equivalent to Timestamp (TS) value. If any of the delayed regulation has a time-delay of fractional unit, the traditional RK4 fails to find the appropriate data (shown in Figure 3(a))). However, the microarray is already stretched with 10 times more values than actual, hence considering the value of h to 10 times smaller than the actual h can mitigate the problem. Figure 3(b) shows that, a fractional time-delayed data is possible to retrieve. At the end of generating the time response for T$\times$ 10 samples, we only consider the samples which has the interval value multiple to 10.

2.4 Reverse engineering GRN with TDSS

The Trigonometric Differential Evolution (TDE) method has been employed to reverse engineer the genetic network with the new TDSS model. This method begins with a knowledge based papulation initialization algorithm, proposed in [5], modified accordingly to incorporate delay of the interactions. The integrity of the individuals are calculated with a newly proposed fitness function. Iteratively, crossovers and mutations of TDE within a population are performed and the best fitting individuals are taken in to account for the future generation. All the experiments are executed for 1000 generations and the TDE parameters are initialized with the initial values considered in [5]. Within the optimization, we have used the Hill Climbing Local Search (HCLS) [3] to every individuals and a flip operation [4] over 5% individuals in the first 300 generations for better mating of parameters. A new Hill Climbing Local Search is also proposed in accordance with delay parameters. Similar to [3–5, 11], we have used the decoupled version of TDSS that infers the parameters of one gene at a time.

2.4.1 Initial population generation with delay

The existing algorithms [6, 11, 14] initialize the P individuals of the first generation randomly whereas our proposed algorithm initializes them with a new Population Initialization (PI) approach operated by dividing

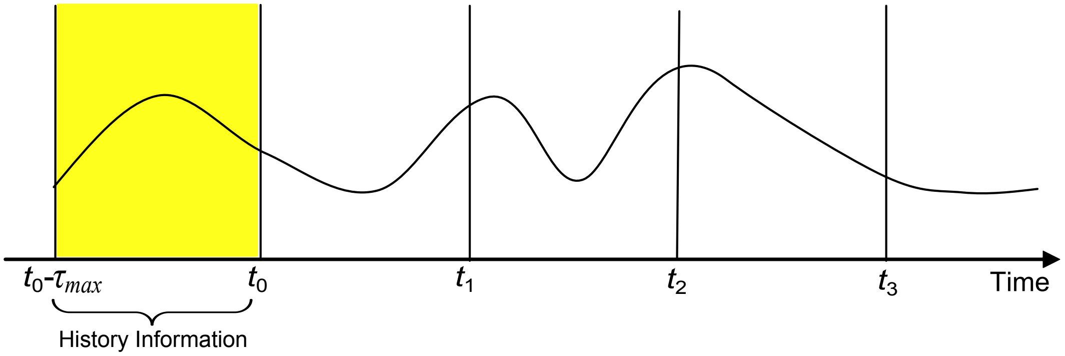

(a)

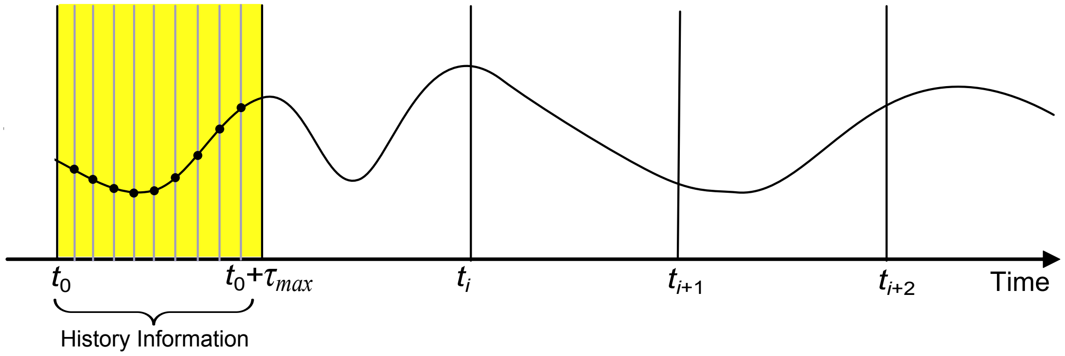

(b)

Figure 2: (a) Gene expression profile for a gene with maximum τ_{max} delay in the interactions with region $[t_{0-\tau_{max}}, t_0]$ as history information (b) Corresponding mapping of $[t_{0-\tau_{max}}, t_0]$ region to $[t_0, t_{0+\tau_{max}}]$, where the region $[t_0, t_{0+\tau_{max}}]$ is now considered as 'History Information'

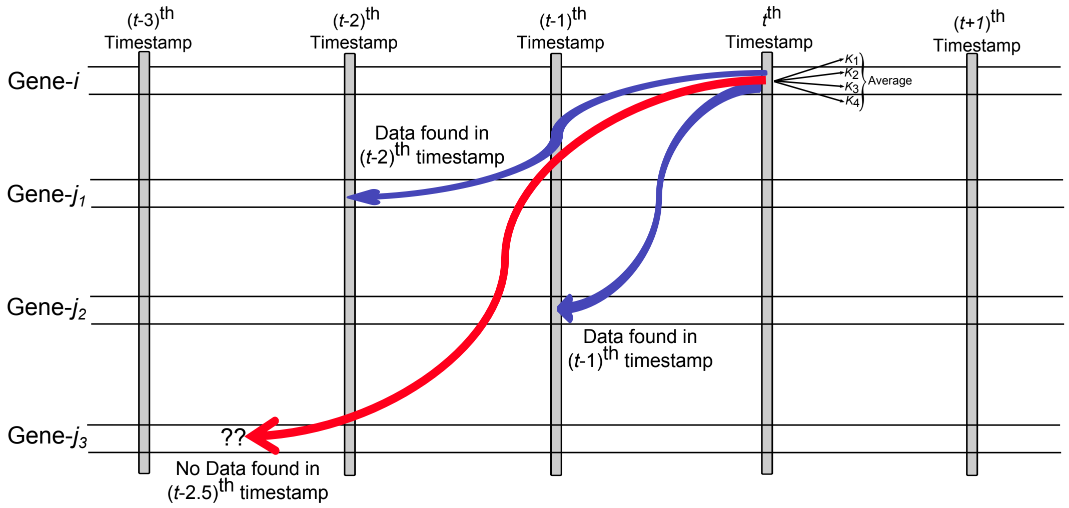

(a)

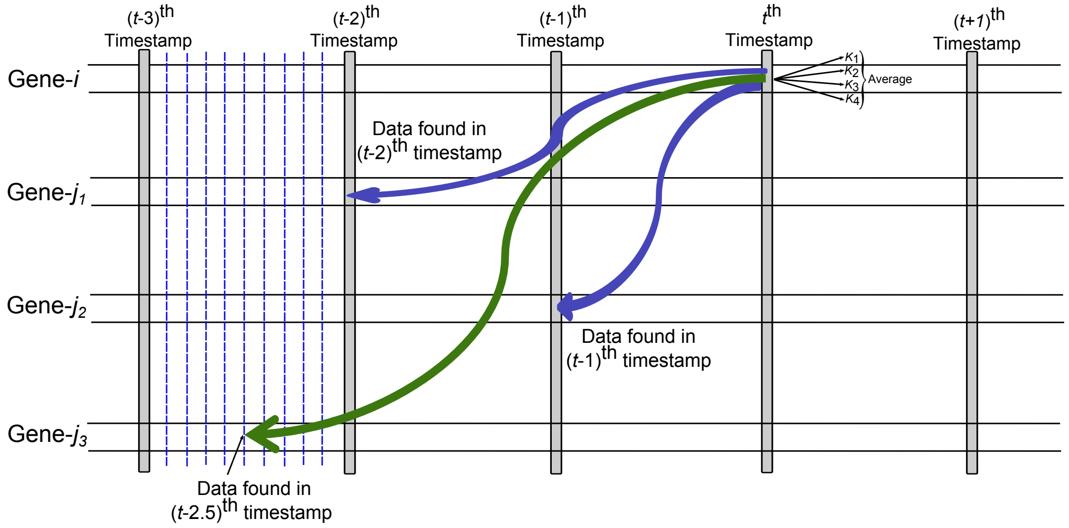

(b)

Figure 3: Data Generation technique in the TDSS where gene-i has incoming interactions of different delays. (a) TDSS with the numerical integration discussed so far does not find any data for $(t-2.5)^{th}$ timestamp from t^{th} timestamp, as the samples are taken in every 1 second (considering 1TS = 1 second) (b) Data found in $(t-2.5)^{th}$ timestamp from t^{th} timestamp when samples are taken with small h value (0.1 sec in the example)

the gene network into two groups: (i) genes with no regulation (zero in-degree), and (ii) genes with one or more regulations (non-zero in-degree). We initialize 90% individual of the population with exactly I regulations (non-zero parameters) and $2N$-I non-regulations (zero valued parameters). The remaining 10% individuals are initialized with zero regulations. This hybrid initialization in the initial population will increase the chance of inferring the genes having zero in-degree correctly and very quickly. In the true biological network, several genes may have zero in-degree, which may not be inferred properly by existing algorithms when initial populations are initialized randomly or considering the max in-degree concept. Because of initialing the population with proposed technique, the optimization converges quickly if the considered genes a true isolated gene. However, adding 90% individuals with I regulations, the small number of zero in-degree genes in the populations would not affect convergence speed and accuracy. In order to initialize the delay parameters for each regulation, we use the knowledge generated in subsection 2.2 using improved PCC. We modify our previously proposed initialization algorithm for any gene-i according to the following steps:

Algorithm 1: *Population Initialization With Delay*(I)

**Step 1:**	Randomly Select 10% individuals
**Step 2:**	Initialize the *kinetic order* values of these individuals with 0
**Step 3:**	Initialize the delay vectors (τ_i^g and τ_i^h) of these individuals with 0
**Step 4:**	Initialize *rate constants* of these individuals randomly
**Step 5:**	**For** each of the remaining individuals **do**
**Step 6:**	Randomly select $2N$-I *kinetic order* values and initialize them to 0
**Step 7:**	**For** each of the remaining I *kinetic order* values **do**
**Step 8:**	Initialize the *kinetic order* value with random value
**Step 9:**	Initialize the corresponding lag values with the knowledge from PCC
**Step 10:**	**End For**
**Step 11:**	Initialize the *rate constants* with random values
**Step 12:**	**End For**

If a node is a true isolated gene, the individual with zero regulations will converge very quickly, with the adjustment of *rate constant* parameters, other than the individuals with I regulations.

2.4.2 New fitness function

In Section 3 of the original document, we propose a new fitness function Adaptive Squared Regulative Error (ASRE) by adding a model complexity term along with SRE term (Eqn. (3) of original document). The model complexity term takes the form of a power law and inverse power law function designed based on the scale-free property. The penalty term divides the entire search space into three regions, as shown in Figure 4. We penalize an individual when the total number of regulation (r_i) lies outside the [J,I] region (Region 1 and Region 3 of Figure 4). That is, when r_i is within the [J,I] range (Region 2 of Figure 4), ASRE avoids penalizing the solution, which in turn, encourage the individual to adjust the r_i interactions to minimize the overall fitness value. In contrast to our previously proposed penalty function [5] (Eqn. (5) in the original document), the C_i produces a symmetric value in both penalty region (Region 1 and Region 3). Although, the multiplication of C_i with $\frac{2N}{2N-r_i}$ violates this symmetric property, nevertheless it strongly discourages the solution for taking more than I regulations.

Other than the penalty terms used in the research work [2–4], we use both in-degrees (J and I) in our new fitness function. Moreover, rather than a static value for I, we adaptively update the values of I and J (discussed in the next subsection), that dynamically narrows down the overall search space.

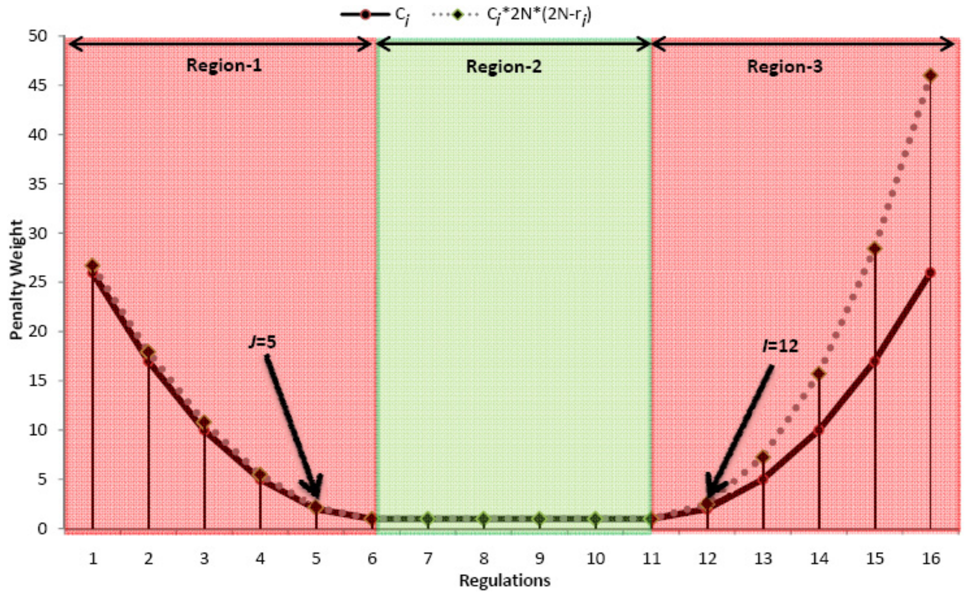

Figure 4: Formulation of penalty function of Eqn. (12) of the original document. Region-1 and Region-3 indicates penalty region for $r_i \leq J$ and $r_i \geq J$, respectively. Curves for C_i and $C_i \times 2N/(2N - r_i)$ are shown with solid and dotted lines, respectively

2.4.3 Adaptive regulatory genes cardinality

We have already introduced the term J, minimum in-degree of a node. As we are working with a decoupled system, we consider I and J separately for each gene. For penalizing the individuals outside the I and J boundary, the individuals are expected to be changed by the number of regulations in every generation. The value of the J is initialized with 0 which allows the system to have zero regulations. The value of J is updated based on the r_i values of each individual after a specified number of iterations. On the other hand, in a limiting case, when all the N nodes are regulating the ith gene, the sub-network of this gene can have at most N regulations (including self-regulations) and, therefore, we initialize the I to N. Similarly, we update the value of I after specified number of iterations based on statistical measurement performed on the individuals of the population at that stage. After every l^{th} iterations, the min in-degree and max in-degree values are recalculated for every current individual. We then update the value of I and J to the minimum among the min in-degrees and the maximum among the max in-degrees within the current population, respectively. After every l^{th} iterations, the search process executes steps of the following algorithm:

**Algorithm 2**: *Adaptive Regulatory Genes Cardinality(ARGC)*	
**Step 1:**	$Max_I = Max(Ind_1, Ind_2, \ldots, Ind_p)$
**Step 2:**	$Min_J = Min(Ind_1, Ind_2, \ldots, Ind_p)$
**Step 3:**	**if** $Max_I < I$ **then**
**Step 4:**	I=Max_I
**Step 5:**	**End if**
**Step 6:**	**if** $Min_J > J$ and $Min_J < I$ **then**
**Step 7:**	J=Min_J
**Step 8:**	**End if**

2.4.4 Hill climbing local search for delay parameters

A Hill-Climbing Local Search (HCLS) algorithm, proposed by Noman et al. [3, 9, 11], were applied in our previous methods [4, 5]. The algorithm is applied over an individual and continues for all P individuals (where P is the total number of individuals) of the population. HCLS first sorts the absolute values of $2N$ kinetic parameters of an individual in non-descending order. Then, making the first kinetic value of this sorted list to zero, the algorithm checks the fitness value. Comparing with previous fitness value, the list is updated with the better fitness value. This technique continues for remaining $2N$-1 kinetic values. The algorithm actually attempts to round-off all the values to zero which are close to zero. In conjunction with the HCLS algorithm [11], we apply our proposed hill climbing algorithm that is suitable for delay parameters (lag values). As the lag values are non-negative, in our proposed Hill Climbing Local Search algorithm for delay parameters (HCLS4lags), we sort the lag values in their ascending order and apply the similar testing that was applied in HCLS. It is considered that, very few regulations are time delayed and hence most of the regulations are instantaneous (lag value is zero). Applying our propose HCLS4lags algorithm, we test whether the low valued delayed regulations are rather instantaneous or a true delayed regulations. The algorithm works according to the following steps:

**Algorithm 3**: *HCLS4Lags(Indiv)*	
**Step 1:**	Sort all the lag values (τ_i^g and τ_i^h) in ascending order. (That is, $Lag(i) \leq Lag(i+1)\ \forall\ i = 1, \ldots, 2N-1$)
**Step 2:**	**For** i=1 to $2N$ **do**
**Step 3:**	Generate $Indiv'$ from $Indiv$ by setting $Lag(i) = 0$
**Step 4:**	**If** $f(Indiv') \leq f(Indiv)$ **then**
**Step 5:**	$Indiv = Indiv'$
**Step 6:**	**End If**
**Step 7:**	**End For**
**Step 8:**	**return** $Indiv$

2.4.5 Multistage refinement algorithm

In the second and final phase of the optimization, we apply the Multistage Refinement Algorithm (MRA), which is especially suitable to noisy time series data; but works equally well for noise free data. It should be noted that, noise is inevitable in microarray data and so the inferred network will consist of few false regulations due to higher noise level. Moreover, because of using the decoupled technique, it may not be possible to infer a network which is completely identical to the target network due to information loss during decoupling. In addition, delayed regulations impact on the expression profile of the corresponding genes and hence they can be considered as noise as well. The multistage refinement algorithm, consists of two stages of Refinement Algorithm [4], in the first stage, takes the P solutions from the first phase of the optimization. Although each of the P individuals is representing the skeletal network, only the individual with minimum fitness value is considered as the candidate solution. However, all the P individuals, including the candidate solution, may contain incorrect regulations for three aforementioned reasons. These incorrect regulations are considered as false regulations and it is necessary to eliminate as many of these as possible from all individuals. The MRA will identify these regulations from all P individuals based on maximum consensus. If more than a specified percentage (δ, in our case 90%) of P individuals of corresponding kinetic value is less than a threshold value ψ (typically between 0.1 and 0.25), then the kinetic value is reset to zero. This resetting technique continues for the remaining ($2N$-1) parameters. After setting zero to these designated regulations (in all individuals), the algorithm executes for a further 250 generations treating these individuals as initial individuals. This time, we avoid executing any local search algorithm by allowing the optimization to adjust the "to be true regulations" to their optimal value. However, because of forced assignment of zero to "probable noise prone" regulations, it is possible that the fitness values of the individuals may increase from its previous values (before change). From the experiment, reported in [4,5] and also in the next section,

this relegation of fitness value is very little (25% of the previous value, i.e. before applying RA). The steps of the RA is shown below in Algorithm 4.

**Algorithm 4**: *Refinement Algorithm (Indiv[1..P])*	
**Step 1:**	**For** i=1 to $TotalKineticOrders$ **do**
**Step 2:**	$Count$=0
**Step 3:**	**For** j=1 to P **do**
**Step 4:**	**If** $Indiv[j].Kinetic[i] < \psi$ **then**
**Step 5:**	$Count = Count$+1
**Step 6:**	**End if**
**Step 7:**	**End For**
**Step 8:**	**If** $Count > (P/100) * \delta$ **then**
**Step 9:**	**For** j=1 to P **do**
**Step 10:**	$Indiv[j].Kinetic[i]$=0
**Step 11:**	**End For**
**Step 12:**	**End If**
**Step 13:**	**End For**

However, there are still possibility that some regulations yet survive with a regulatory weight less than ψ at the end of the first phase of refinement algorithm (Algorithm 4). This situation was validated with appropriate example in [4]. That is why, we apply another run of the RA by taking the individuals from the first stage run of RA, hence we call it Multistage Refinement Algorithm (MRA). It has been found that, refinement algorithm [4] and subsequently multistage refinement algorithm [5] improve the performance of the inferred network in terms of specificity and average error.

3 Additional experimental results and discussions

In order to prove the supremacy of the proposed method over existing methods, we considered two synthetic networks and two real-life biological networks. The 5-gene synthetic network, as shown in Table 1 of the original document, was widely used by most of the S-System based methods. We have considered three different configurations; Configuration-1 (Conf-1), which is the actual network with instantaneous regulations only, Configuration-2 (Conf-2), that contains 5 delayed regulation of equal weight and 8 instantaneous regulations, Configuration-3 (Conf-3) has 6 delayed regulations of various weight and 7 instantaneous regulations, as shown in Table 2 of the original document. Considering $\tau_{max} = 3TS$, the time responses are generated and shown in Figure 5. Although, the responses for the gene-2 and gene-3 in delayed configurations (Conf-2 and Conf-3) are close to the original response (Conf-1), they substantially differs for three other genes, which surely affect the reverse engineering process for traditional (no-delay) S-System modeling. As a result, existing techniques failed to infer the original network with 100% accuracy. However, TDSS successfully inferred all the regulatory parameters very accurately along with rate constants, as shown in Table 1. Moreover, TDSS inferred the delay matrices precisely with minimum error, as it can be observed from Table 2. For any delayed regulation, inferred lag value with ±0.1TS is considered as minimum error while 0.1TS as lag value for instantaneous regulations are considered as minimum error. The lag values for the regulations falsely inferred as delayed regulations having minimum error are shown with red mark in Table 2.

We have calculated the sensitivity (S_n), specificity (S_p), precision (P_r), and F-score (F) of the networks

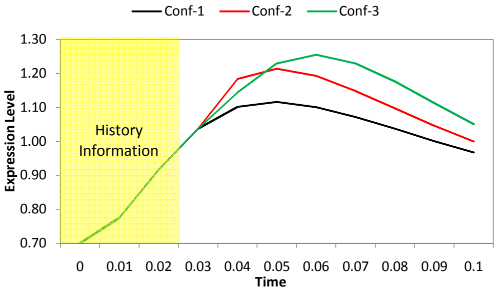

(a) Gene-1

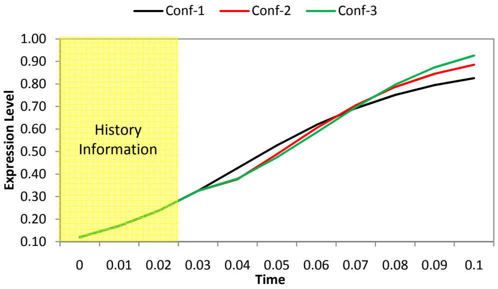

(b) Gene-2

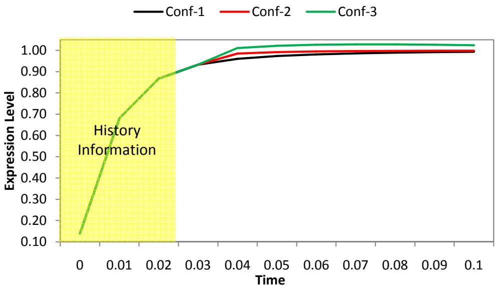

(c) Gene-3

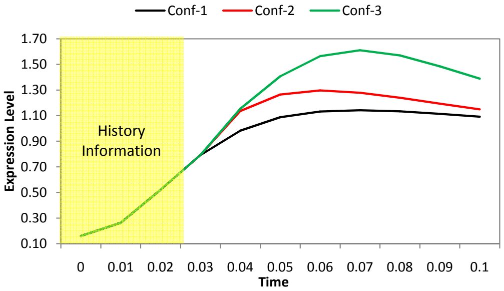

(d) Gene-4

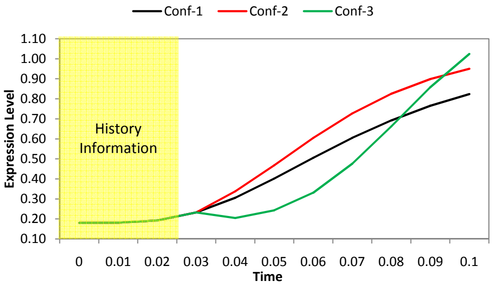

(e) Gene-5

Figure 5: Time responses for all 5 genes in three configurations with $\tau_{max} = 3$TS

inferred by TDSS and three existing methods [3, 5, 15] according to the following equations:

$$
\begin{aligned}
S_n &= \frac{TP}{TP + FN} \\
S_p &= \frac{TN}{TN + FP} \\
P_r &= \frac{TP}{TP + FP} \\
F &= \frac{2(P_r * S_n)}{P_r + S_n}
\end{aligned}
\tag{15}
$$

Here, TP (True Positive) represents the number of regulations correctly predicted by the inference algorithm, TN (True Negative) represents the number of non-regulations correctly predicted as non-regulations, FP (False Positive) indicates the number of regulations falsely predicted by the inference algorithm, and FN

Table 1: Inferred parameters for three configurations by TDSS

	G#	α_i	$g_{i,1}$	$g_{i,2}$	$g_{i,3}$	$g_{i,4}$	$g_{i,5}$	β_i	$h_{i,1}$	$h_{i,2}$	$h_{i,3}$	$h_{i,4}$	$h_{i,5}$
Inferred (Conf-1)	1	5.03	0.00	0.00	0.98	0.00	-1.00	10.04	1.99	0.00	0.00	0.00	0.00
	2	9.93	2.00	0.00	0.00	0.00	0.00	9.92	0.00	1.99	0.00	0.00	0.00
	3	10.03	0.00	-1.00	0.00	0.00	0.00	10.03	0.00	-1.00	1.99	0.00	0.00
	4	8.01	0.00	0.00	2.02	0.00	-1.01	10.02	0.00	0.00	0.00	2.01	0.00
	5	10.01	0.00	0.00	0.00	2.10	0.00	10.01	0.00	0.00	0.00	0.00	2.00
Inferred (Conf-2)	1	5.00	0.00	0.00	0.91	0.00	-0.96	9.56	1.99	0.00	0.00	0.00	0.00
	2	10.10	1.99	0.00	0.00	0.00	0.00	9.90	0.00	1.94	0.00	0.00	0.00
	3	9.93	0.00	-1.00	0.00	0.00	0.00	9.94	0.00	-0.98	2.03	0.00	0.00
	4	7.99	0.00	0.00	1.99	0.00	-1.00	10.01	0.00	0.00	0.00	2.01	0.00
	5	10.01	0.00	0.00	0.00	2.00	0.00	10.05	0.00	0.00	0.00	0.00	2.01
Inferred (Conf-3)	1	5.01	0.00	0.00	1.01	0.00	-0.99	9.88	1.95	0.00	0.00	0.00	0.00
	2	10.05	1.99	0.00	0.00	0.00	0.00	9.95	0.00	1.98	0.00	0.00	0.00
	3	9.98	0.00	-1.00	0.00	0.00	0.00	9.79	0.00	-1.03	2.00	0.00	0.00
	4	8.16	0.00	0.00	1.91	0.00	-1.01	10.21	0.00	0.00	0.00	2.06	0.00
	5	10.04	0.00	0.00	0.00	2.00	0.00	10.10	0.00	0.00	0.00	0.00	2.01

G# indicates the Gene number

(False Negative) indicates number of regulations missed by the algorithm. The results for all three configuration with and without the presence of noise are shown in Table 3 and Table 4 in the original document. In addition, we derive the following equation to obtain the average error for delay parameters ($E^*(\tau)$):

$$E^*(\tau) = \frac{1}{P_s(\tau)Q} \sum_{k=1}^{Q} \sum_{i=1}^{P_s(\tau)} |d_{k,i,1}(\tau) - d_{k,i,2}(\tau)| \qquad (16)$$

Here, Q is the number of runs (=5 in our experiment) and $P_s(\tau)$ is the total number of lag values estimated in single run (=$2 \times N \times N$). $d_{k,i,1}(\tau)$ and $d_{k,i,2}(\tau)$ indicate the ith target and estimated delay (lag) parameters in k^{th} run, respectively. According to Eqn. (16), we obtain the $E^*(\tau)$ as 0.012, 0.014, 0.018 for Conf-1, Conf-2, Conf-3 in TDSS, respectively. It can be observed that, few instantaneous regulations are inferred as delayed regulation with minimum error (0.1TS), which might be due to increase of number of parameters in the TDSS and approximation for decoupling. Moreover, we put emphasis on inferring the regulators in the fitness function and only single local search was invoked for optimizing delay parameters. Further attention on including delay parameters in the fitness function may infer the network more accurately with zero value for $E^*(\tau)$. In order to show the accuracy of the proposed TDSS, we have shown the evaluations of time-responses of one common gene for all three configurations in Sec. 4.1 of the original document. In this section, we present time-responses for one more gene for each configuration in Fig. 6-8. We can observe that, the time-responses generated by the proposed method are very close to the original time-expression and follow the trends of the target expression patterns.

The performances for the medium scale network was also excellent for TDSS. For noise free data, the proposed TDSS missed inferring only one regulation in Conf-5. The inferred system parameters for genes having true delayed regulations, along with corresponding their lag values for Conf-4 and Conf-5 are shown in Table 3 and Table 4, respectively. Figure 9 and Figure 10 show the time-expressions for two selective genes with four different levels of noise in data.

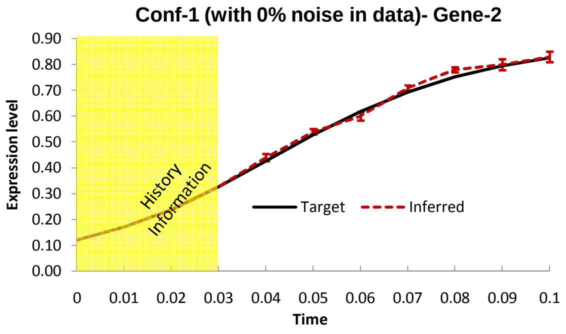

(a) *0% Noise in Data*

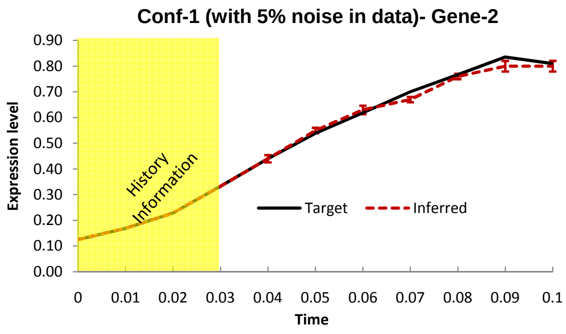

(b) *5% Noise in Data*

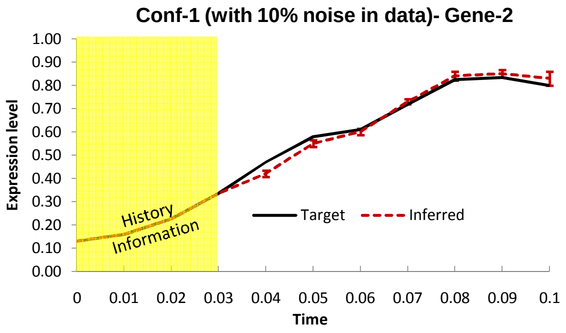

(c) *10% Noise in Data*

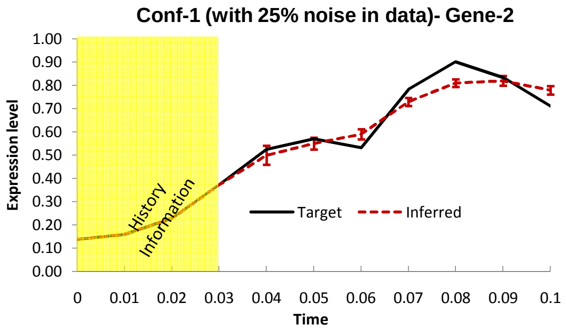

(d) *25% Noise in Data*

Figure 6: Dynamics for Gene-2 of Conf-1. Solid lines and dotted lines indicate respectively target and inferred (by TDSS) time-expressions in (a) Noise free data (b) 5% Noise in data (c) 10% Noise in data (d) 25% Noise in data. The yellow shaded region indicates the history information and the error bars indicate 95% confidence interval.

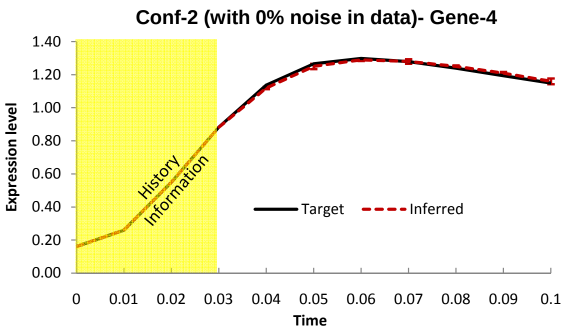

(a) *0% Noise in Data*

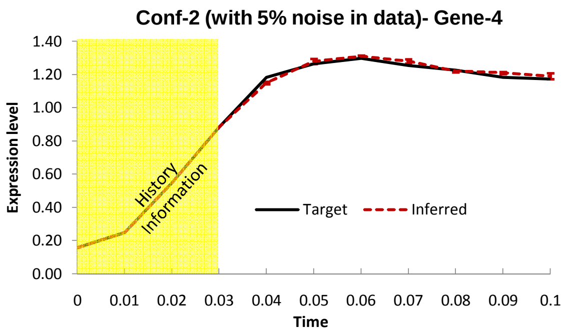

(b) *5% Noise in Data*

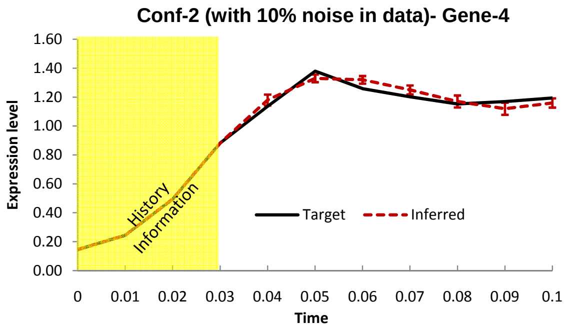

(c) *10% Noise in Data*

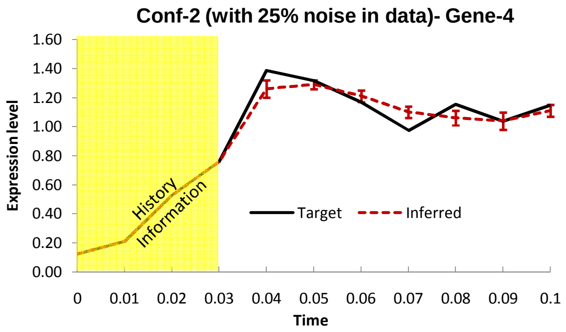

(d) *25% Noise in Data*

Figure 7: Dynamics for Gene-4 of Conf-2. Solid lines and dotted lines indicate respectively target and inferred (by TDSS) time-expressions in (a) Noise free data (b) 5% Noise in data (c) 10% Noise in data (d) 25% Noise in data. The yellow shaded region indicates the history information and the error bars indicate 95% confidence interval.

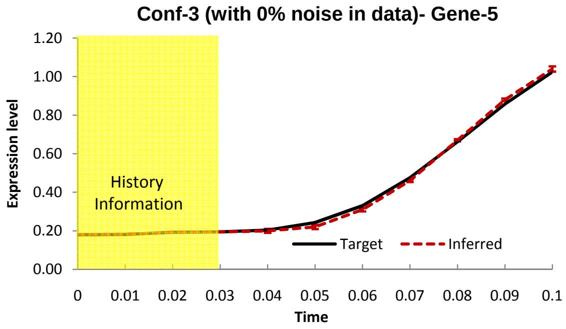

(a) *0% Noise in Data*

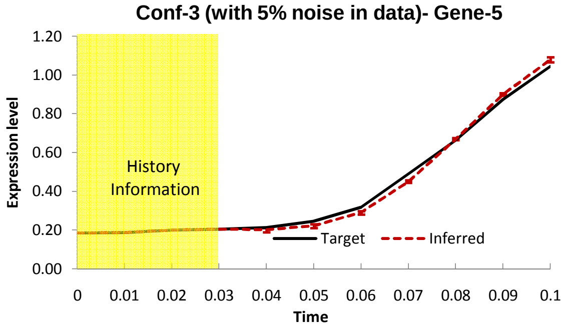

(b) *5% Noise in Data*

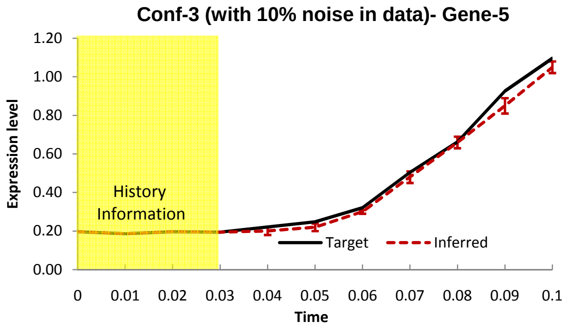

(c) *10% Noise in Data*

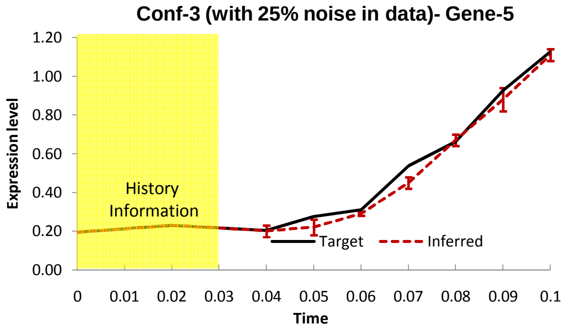

(d) *25% Noise in Data*

Figure 8: Dynamics for Gene-5 of Conf-3. Solid lines and dotted lines indicate respectively target and inferred (by TDSS) time-expressions in (a) Noise free data (b) 5% Noise in data (c) 10% Noise in data (d) 25% Noise in data. The yellow shaded region indicates the history information and the error bars indicate 95% confidence interval.

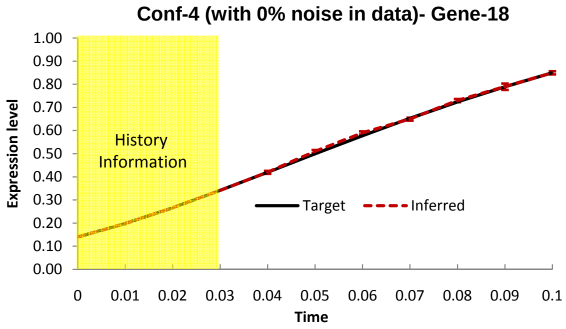

(a) *0% Noise in Data*

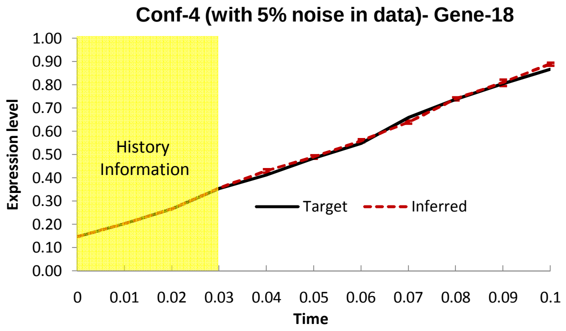

(b) *5% Noise in Data*

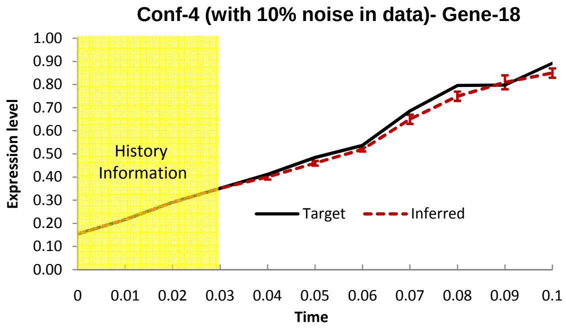

(c) *10% Noise in Data*

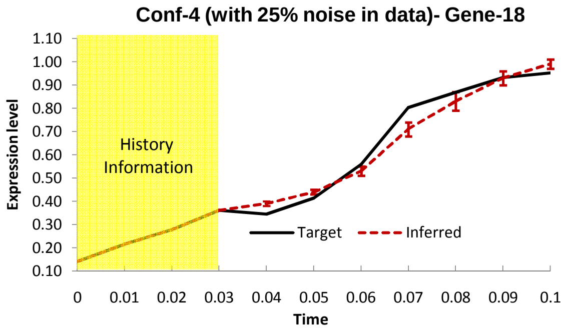

(d) *25% Noise in Data*

Figure 9: Dynamics for Gene-18 of Conf-4. Solid lines and dotted lines indicate respectively target and inferred (by TDSS) time-expressions n (a) Noise free data (b) 5% Noise in data (c) 10% Noise in data (d)25% Noise in data. The yellow shaded region indicates the history information and the error bars indicate 95% confidence interval.

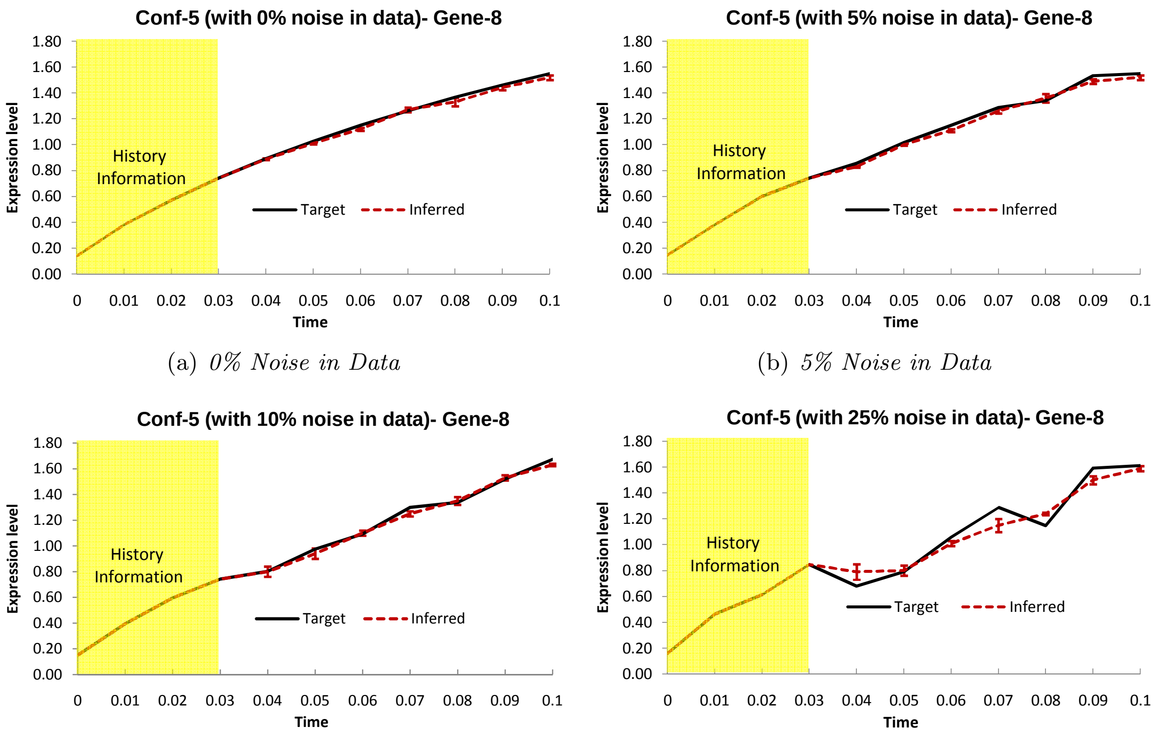

(a) *0% Noise in Data*

(b) *5% Noise in Data*

(c) *10% Noise in Data*

(d) *25% Noise in Data*

Figure 10: Dynamics for Gene-8 of Conf-5. Solid lines and dotted lines indicate respectively target and inferred (by TDSS) time-expressions in (a) Noise free data (b) 5% Noise in data (c) 10% Noise in data (d)25% Noise in data. The yellow shaded region indicates the history information and the error bars indicate 95% confidence interval.

Table 2: τ^g and τ^h matrices inferred by TDSS for three configurations

		τ^g					τ^h				
	G#	$\tau^g_{i,1}$	$\tau^g_{i,2}$	$\tau^g_{i,3}$	$\tau^g_{i,4}$	$\tau^g_{i,5}$	$\tau^h_{i,1}$	$\tau^h_{i,2}$	$\tau^h_{i,3}$	$\tau^h_{i,4}$	$\tau^h_{i,5}$
Conf-1	1	0.0	0.0	0.0	0.0	0.0	**0.1**	0.0	0.0	0.0	0.0
	2	**0.1**	0.0	0.0	0.0	0.0	0.0	**0.1**	0.0	0.0	0.0
	3	0.0	**0.1**	0.0	0.0	0.0	0.0	0.0	0.0	0.0	0.0
	4	0.0	0.0	0.0	0.0	0.0	0.0	0.0	0.0	**0.1**	0.0
	5	0.0	0.0	0.0	0.0	0.0	0.0	0.0	0.0	0.0	**0.1**
Conf-2	1	0.0	0.0	0.0	0.0	**0.9**	**0.1**	0.0	0.0	0.0	0.0
	2	**1.0**	0.0	0.0	0.0	0.0	0.0	**0.1**	0.0	0.0	0.0
	3	0.0	**1.1**	0.0	0.0	0.0	0.0	**1.0**	0.0	0.0	0.0
	4	0.0	0.0	0.0	0.0	**0.9**	0.0	0.0	0.0	**0.1**	0.0
	5	0.0	0.0	0.0	0.0	0.0	0.0	0.0	0.0	0.0	**0.1**
Conf-3	1	0.0	0.0	**1.2**	0.0	0.0	**0.1**	0.0	0.0	0.0	0.0
	2	**1.2**	0.0	0.0	0.0	0.0	0.0	**0.1**	0.0	0.0	0.0
	3	0.0	**1.2**	0.0	0.0	0.0	0.0	**0.9**	0.0	0.0	0.0
	4	0.0	0.0	0.0	0.0	**1.0**	0.0	0.0	0.0	**0.1**	0.0
	5	0.0	0.0	0.0	**2.2**	0.0	0.0	0.0	0.0	0.0	**0.1**

G# indicates the Gene number

References

1. Kikuchi S, Tominaga D, Arita M, Takahashi K, Tomita M: **Dynamic modeling of genetic networks using genetic algorithm and S-system**. *Bioinformatics* 2003, **19**(5):643–650.

2. Kimura S, Ide K, Kashihara A, Kano M, Hatakeyama M, Masui R, Nakagawa N, Yokoyama S, Kuramitsu S, Konagaya A: **Inference of S-system models of genetic networks using a cooperative coevolutionary algorithm**. *Bioinformatics* 2005, **21**(7):1154–63.

3. Noman N, Iba H: **Inferring Gene Regulatory Networks using Differential Evolution with Local Search Heuristics**. *IEEE Transactions on Computational Biology and Bioinformatics* 2007, **4**:634–647.

4. Chowdhury AR, Chetty M: **An Improved Method to Infer Gene Regulatory Network using S-System**. In *IEEE CEC* 2011:1012–1019.

5. Chowdhury AR, Chetty M, Vinh XN: **Adaptive Regulatory Genes Cardinality for Reconstructing Genetic Networks**. In *IEEE CEC* 2012:955–962.

6. Noman N, Iba H: **On the Reconstruction of Gene Regulatory Networks from Noisy Expression Profiles**. In *IEEE CEC* 2006:2543–2550.

7. Akaike H: **Information theory and an extension of the maximum likelihood principle**. In *Second Int. Symposium on Information Theory* 1973:267–281.

8. Storn R, Price KV: **Differential evolution - a simple and efficient heuristic for global optimization over continuous spaces**. *Journal of Global Optimization* 1997, **11**:341–359.

9. Noman N, Iba H: **Inference of genetic networks using S-system: information criteria for model selection**. In *GECCO* 2006:263–270.

Table 3: Parameters inferred by TDSS for few genes of Conf-4.

Genes	Inferred parameter values
Gene-3	α_3=10.14, β_3=10.09, $g_{3,15} = -0.73$, $\tau^g_{3,15} = 0.1$, $h_{3,3} = 1.1$, $\tau^h_{3,3} = 0.1$
Gene-5	α_5=9.99, β_5=10.02, $g_{5,1} = 1.02$, $\tau^g_{5,1} = 0.0$, $h_{5,5} = 0.9$, $\tau^h_{5,5} = 0.1$
Gene-7	α_7=10.11, β_7=10.07, $g_{7,2} = 1.18$, $g_{7,3} = -0.85$, $g_{7,10} = 1.62$, $\tau^g_{7,2} = 0.0$, $\tau^g_{7,3} = 0.1$, $\tau^g_{7,10} = 0.1$, $h_{7,7} = 1.03$, $\tau^h_{7,7} = 0.1$
Gene-10	α_{10}=9.98, β_{10}=10.02, $g_{10,6} = -0.27$, $g_{10,14} = 1.02$, $\tau^g_{10,6}$ =0.0, $\tau^g_{10,14}$ =0.1, $h_{10,10} = 1.04$, $\tau^h_{10,10} = 0.1$
Gene-14	α_{14}=9.91, β_{14}=10.11, $g_{14,11} = -0.38$, $\tau^g_{14,11} = 0.0$, $h_{14,14} = 1.11$, $\tau^h_{14,14} = 0.1$
Gene-18	α_{18}=10.10, β_{18}=10.09, $g_{18,14} = 1.2$, $\tau^g_{18,14} = 0.1$, $h_{18,18} = 1.4$, $\tau^h_{18,18} = 0.1$
Gene-19	α_{19}=9.95, β_{19}=10.10, $g_{19,12} = 1.48$, $g_{19,17} = 0.68$, $\tau^g_{19,12} = 0.1$, $\tau^g_{19,17} = 0.1$, $h_{19,19} = 0.9$, $\tau^h_{19,19} = 0.0$
Gene-20	α_{20}=10.04, β_{20}=10.03, $g_{20,14} = 1.09$, $g_{20,17} = 1.7$, $\tau^g_{20,14} = 0.1$, $\tau^g_{20,17} = 0.0$, $h_{20,20} = 0.91$, $\tau^h_{20,20} = 0.0$

Table 4: Parameters inferred by TDSS for few genes of Conf-5.

Genes	Inferred parameter values
Gene-3	α_3=9.97, β_3=10.10, $g_{3,15} = -0.72$, $\tau^g_{3,15} = 1.2$, $h_{3,3} = 1.05$, $\tau^h_{3,3} = 0.0$
Gene-5	α_5=10.10, β_5=10.01, $g_{5,1} = 1.04$, $\tau^g_{5,1} = 1.1$, $h_{5,5} = 1.08$, $\tau^h_{5,5} = 0.1$
Gene-7	α_7=10.03, β_7=10.02, $g_{7,2} = 1.25$, $g_{7,3} = -0.83$, $g_{7,10} = 1.69$, $\tau^g_{7,2} = 0.1$, $\tau^g_{7,3} = 0.0$, $\tau^g_{7,10} = 1.6$, $h_{7,7} = 0.97$, $\tau^h_{7,7} = 0.0$
Gene-10	α_{10}=9.99, β_{10}=9.97, $g_{10,6} = -0.27$, $g_{10,14} = 0.98$, $\tau^g_{10,6}$ =2.0, $\tau^g_{10,14}$ =0.1, $h_{10,10} = 1.02$, $\tau^h_{10,10} = 0.0$
Gene-14	α_{14}=10.34, β_{14}=10.18, $g_{14,11} = -0.42$, $g_{14,18} = 0.3$, $\tau^g_{14,11} = 1.4$, $\tau^g_{14,18} = 0.1$, $h_{14,1} = 0.29$, $h_{14,14} = 1.19$, $\tau^h_{14,1} = 0.1$, $\tau^h_{14,14} = 0.2$
Gene-18	α_{18}=9.90, β_{18}=10.05, $g_{18,14} = 1.3$, $\tau^g_{18,14} = 2.0$, $h_{18,15} = 0.26$, $h_{18,18} = 1.1$, $\tau^h_{18,15} = 0.4$, $\tau^h_{18,18} = 0.1$
Gene-19	α_{19}=10.11, β_{19}=10.08, $g_{19,12} = 1.35$, $g_{19,17} = 0.63$, $\tau^g_{19,12} = 0.0$, $\tau^g_{19,17} = 0.6$, $h_{19,19} = 1.08$, $\tau^h_{19,19} = 0.1$
Gene-20	α_{20}=9.89, β_{20}=10.13, $g_{20,14} = 1.07$, $g_{20,17} = 1.57$, $\tau^g_{20,14} = 1.1$, $\tau^g_{20,17} = 0.1$, $h_{20,17} = 0.33$, $h_{20,20} = 1.1$, $\tau^h_{20,17} = 0.2$, $\tau^h_{20,20} = 0.1$

10. Fan HY, Lampinen J: **A trigonometric mutation operation to differential evolution**. *Journal of Global Optimization* 2003, **27**:105–129.

11. Noman N: **A Memetic Algorithm for Reconstructing Gene Regulatory Networks from Expression Profile**. *PhD thesis*, Graduate School of Frontier Sciences at the University of Tokyo 2007.

12. Stigler SM: **Francis Galton's Account of the Invention of Correlation**. *Statistical Science* 1989, **4(2)**:73–79.

13. Press W, Teukolsky S, Vetterling W, Flannery B: *Numerical recipies in C 2nd edition.* Cambridge University Press 1995.

14. Hasan MM, Noman N, Iba H: **A prior knowledge based approach to infer gene regulatory networks**. In *Proceedings of the International Symposium on Biocomputing* 2010:15–17.

15. Yu J, Smith VA, Wang PP, Hartemink AJ, Jarvis ED: **Advances to Bayesian network inference for generating causal networks from observational biological data**. *Bioinformatics* 2004, **20**:3594–3603.
